# Cascading expression of ApiAP2 transcription factors controls daughter cell assembly in *Toxoplasma gondii*

**DOI:** 10.1371/journal.ppat.1012810

**Published:** 2024-12-30

**Authors:** Maanasa Bhaskaran, Venkat Mudiyam, Thomas Mouveaux, Emmanuel Roger, Mathieu Gissot

**Affiliations:** University of Lille, CNRS, Inserm, CHU Lille, Institut Pasteur de Lille, U1019—UMR 9017—CIIL—Center for Infection and Immunity of Lille, Lille, France; University of Geneva Faculty of Medicine: Universite de Geneve Faculte de Medecine, SWITZERLAND

## Abstract

Pathogenesis of *Toxoplasma gondii* in the intermediate host is based on the tachyzoite ability to divide rapidly to produce significant amount of daughter cells in a reduce time frame. The regulation of the cell-cycle specific expression program is therefore key to their proliferation. Transcriptional regulation has a crucial role in establishing this expression program and transcription factors regulates many aspects of tachyzoite cell cycle. We explored the role of two ApiAP2 transcription factors, TgAP2XII-9 and TgAP2III-2, during the cell cycle of the tachyzoite form. While TgAP2III-2 has only a minor impact on the tachyzoite proliferation, we show that TgAP2XII-9 regulates many aspects of the cell cycle including the proper assembly of the daughter cells inner membrane complex and temporal expression of many virulence genes. Creation of a double mutant strain for TgAP2XII-9 and TgAP2III-2 shows that TgAP2XII-9 had a prominent role during daughter cell assembly. Using transcriptomics and Cut&Tag, we demonstrate that TgAP2XII-9 mainly acts through the transcriptional control of at least 300 genes promoters. Interestingly, TgAP2XII-9 plays a crucial role repressing the expression of genes necessary for budding initiation and activating genes necessary for microneme *de novo* formation. We also explored the importance of the AP2 domain of TgAP2XII-9 demonstrating its critical role to exert its function. Therefore, we showed that TgAP2XII-9 is a crucial transcription factor which is key to daughter cell assembly post budding initiation.

## Author summary

*Toxoplasma gondii* is a is a unicellular eukaryotic pathogen that infects a wide range of animals. Pathogenesis of this parasite relies on its ability to rapidly proliferate and disseminate in the host. The *T*. *gondii* tachyzoite is the fast replicating form of the parasite that causes all the symptoms of toxoplasmosis in humans. Replication of this parasite form relies on a simplified and fast cell-cycle that is mainly controlled at the transcriptional level. We studied two transcription factors that bear differential role in regulating the establishment of the cell-cycle dependent expression profiles after initiation of daughter cell formation. We used trancriptomics, CUT&Tag and phenotypic assays to decipher the roles of these ApiAP2 transcription factors. Our data highlight the crucial role of TgAP2XII-9 both as a repressor and activator of subset of genes. This transcription factor activates the expression of virulence factor genes such as micronemes encoding transcripts therefore regulating the biogenesis of these organelles. This study shows that ApiAP2 transcription factors are timely expressed to activate the expression profile needed at each phase of the cell cycle.

## Introduction

*Toxoplasma gondii* is a eukaryotic pathogen classified within the phylum Apicomplexa, which encompasses many protozoan parasites of significant medical and veterinary concern, including *Plasmodium* (the causative agent of malaria) and *Cryptosporidium* (responsible for cryptosporidiosis). *T*. *gondii* has garnered considerable attention as an opportunistic pathogen linked to encephalitis and systemic infections in immunocompromised individuals, particularly those with HIV/AIDS [1]. Approximately one-third of the world’s population is estimated to be infected by *T*. *gondii*. This parasite has the ability to cross the blood-brain barrier and establish a chronic infection by differentiating into a dormant, drug-resistant bradyzoite stage [2]. The rapidly growing tachyzoite form of the parasite is responsible for the clinical manifestations of the disease in humans. The tachyzoite’s ability to proliferate is crucial to its pathogenesis. *T*.*gondii* tachyzoites exhibit an unusual cell cycle characterized by closed mitosis. The cell cycle phases include: G1, S, and M. Up until recently the G2 phase was thought to be short or absent [3]. A recent study by Hawkins et al.,2024 [4] strongly suggested the presence of a short G2 phase that overlaps with S/M phase. Tachyzoites multiply asexually within the host cell through a process called endodyogeny where two daughter parasites form within the parent parasite. The G1 phase of *T*. *gondii* endodyogeny, when canonical housekeeping tasks preparing for the S phase occur, comprises about half of the division cycle. During the S phase, chromosome replication and organelle duplication is coordinated with the initiation of the budding in a process called budding [3]. Centriole duplication occurs early in the S-phase, and budding begins late in the S-phase of the cell cycle. Early on, cortical microtubules and the conoid form near the centrioles, anchored by the apical polar ring (APR), which acts as a microtubule-organizing center [5]. The APR organizes microtubules (MT) growth and establishes apical-basal polarity, essential for structural organization [6]. The basal complex is formed concomitantly to the APR, as TgMORN1 and TgBCC0 are among the initial components deposited within the budding daughter cells, positioned in alignment with the duplicated centrosomes [7–14]. These proteins coordinate cytoskeletal development, with TgBCC0 and other basal complex proteins guiding the five-fold structural symmetry of the daughter cells [15]. The inner membrane complex (IMC) of the daughter cells is generated from the apical pole, beginning with the formation of the apical cap of the IMC, followed by the central IMC and basal IMC sub-compartments [16]. This process first encapsulates the divided Golgi [17], followed by the apicoplast [18] and subsequently nucleus and endoplasmic reticulum [19]. As the daughter parasites mature, the maternal cytoskeleton disintegrates, and the maternal plasma membrane is repurposed onto the emerging daughters [12]. The tachyzoite cell cycle is regulated by both transcriptional and post-translational mechanisms. For instance, transcription factors (TFs) such as TgAP2X-5 and TgAP2XI-5 have been demonstrated to control the cell-cycle-dependent expression of many virulence factors [20]. Cyclin dependent kinases (cdks) and cyclins serve as the primary regulators of the cell cycle, orchestrating progression through various phases. Transcription factors, acting downstream of these regulators, execute specific transcriptional programs in response to Cdk and cyclin signalling [21–23]. AP2 transcription factors function as critical downstream effectors, controlling essential processes like replication and daughter cell budding in response to cell cycle cues. The majority of transcriptional regulators of the tachyzoite cell cycle belong to the plant-like Arabidopsis APETALA-2 (AP-2) family [24,25]. The AP2 domain functions as a DNA-binding domain within AP2 transcription factors, allowing each factor to bind specific promoter regions and regulate gene expression. In *T*. *gondii*, cell cycle-dependent expression profiles can be regulated by the cooperative action multiple AP2 transcription factors. These TFs may act in concert or sequentially at different stages of the cell cycle to fine-tune the expression of target genes essential for cell division and development [20]. The roles of some of the AP2 TFs have been uncovered. For instance, TgAP2XII-8 regulates ribosomal protein production during the early G1 phase [26]. TgAP2IX-5 controls the initial steps of budding [27] by regulating the expression of hundreds of genes, including those coding for elements destined for early incorporation into the developing daughter buds, such as apical cap proteins TgAC2, TgAC7, and TgISP1 and budding markers such as TgIMC1, TgIMC3, TgIMC4, and TgIMC10. Additionally, TgAP2IX-5 directly controls the expression of other TFs, notably TgAP2III-2, TgAP2XII-2, and TgAP2XII-9. TgAP2XII-2 is essential for the proper progression through S-phase [28] indicating that TgAP2IX-5 controls the expression of TFs that may be essential for the continuation of the cell cycle. The roles of TgAP2III-2 and TgAP2XII-9 remain unexplored.

In this study, we functionally characterized two cell-cycle-dependent ApiAP2 TFs, TgAP2XII-9 and TgAP2III-2, whose expression is directly regulated by TgAP2IX-5. While TgAP2III-2 had non-essential roles during the tachyzoite cell cycle, we demonstrate that TgAP2XII-9 is crucial for proper formation of daughter parasites after budding initiation. TgAP2XII-9 acts as a repressor of a subset rhoptries, rhoptry neck proteins, and IMC apical cap genes. Conversely, it activates the expression of a set of genes encoding for micronemes and dense granule proteins. Thus, TgAP2XII-9 is a critical TF that controls gene expression during the daughter cell completion and the de novo formation of virulence organelles.

## Results

### TgAP2XII-9 and TgAP2III-2 are cell-cycle regulated and expressed during the S/M phase

**Fig 1:**
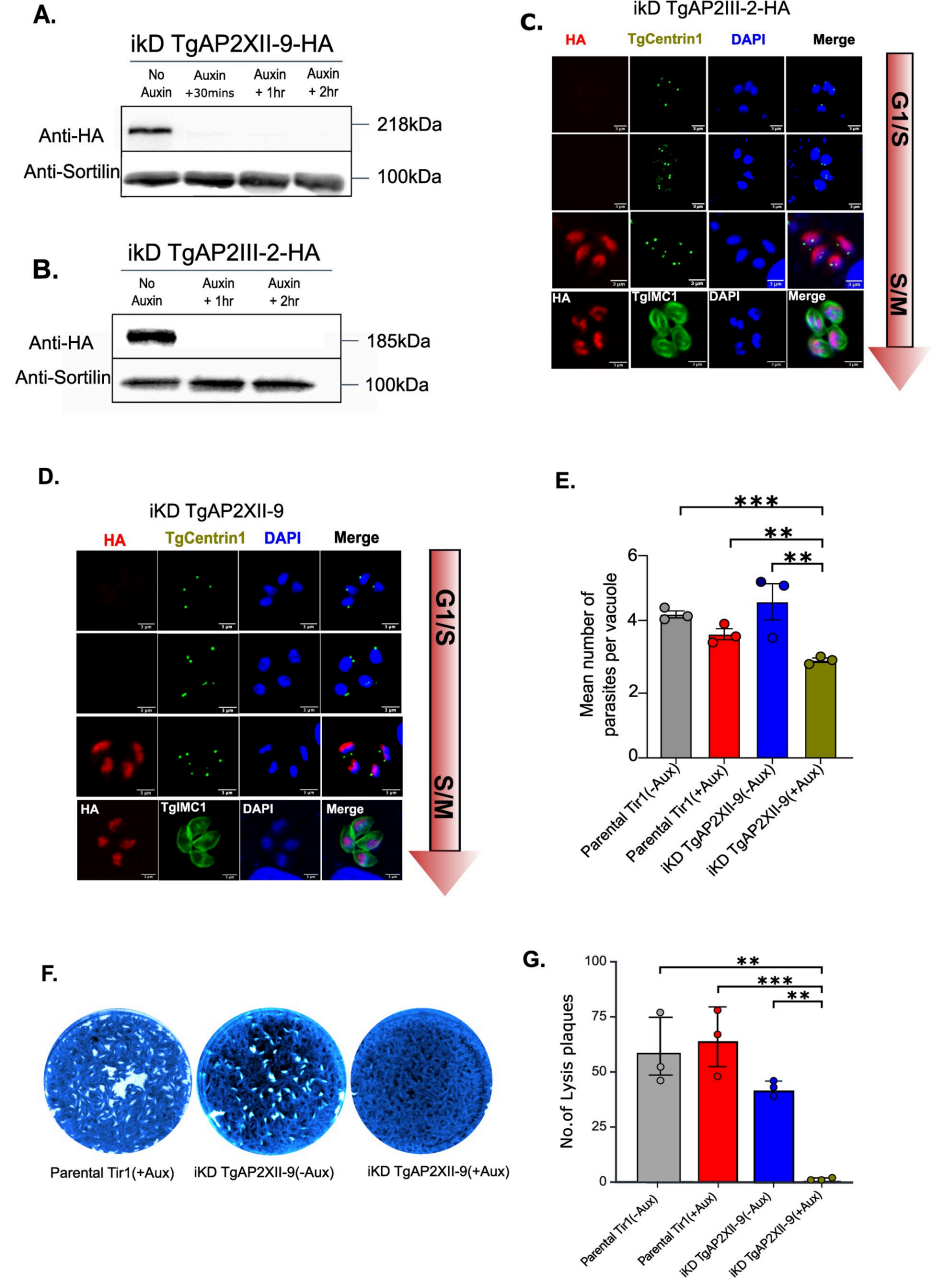
TgAP2XII-9 and TgAP2III-2 are cell-cycle regulated and expressed during the S/M phase. **TgAP2XII-9 is essential for growth and proliferation *in vitro* (A, B)** Western blot analysis of total protein extracts from parental and iKD TgAP2XII-9 and TgAP2III-2 strains treated with Auxin for varying durations. The blots were probed with anti-HA to detect TgAP2XII-9 and TgAP2III-2 protein levels (upper panel) and with anti-TgSortilin as a normalization control (lower panel), validating the AID system. **(C, D)** Cell cycle expression of TgAP2III-2 and AP2XII-9 as realized by IFA using anti-HA antibody and cell cycle markers TgCentrin1 and TgIMC1. Scale bar = 3 μm **(E)** Growth assay for parental and iKD TgAP2XII-9 strains with and without 24-hour auxin treatment. Statistical analysis was performed using a two-tailed Student’s t-test, with significance indicated by ***p < 0.001, *p<0.05, Data are presented as mean ± s.d. (n  =  3). **(F)** Plaque assay depicting proliferation and growth of Parental and iKD AP2XII-9 strains with and without auxin for 7 days. **(G)** Quantification of the no.of lysis plaques for parental and iKD TgAP2XII-9 strains. Statistical analysis was performed using a two-tailed Student’s t-test, with significance indicated by **p < 0.01, *p<0.05, Data are presented as mean ± s.d. (n  =  3).

The genome wide CRISPR screening performed by Sidik *et al*. in 2016 [29] allows the identification of genes essential for tachyzoite growth in the *T*. *gondii* genome. Genes with negative fitness scores are considered essential for the parasite’s fitness [29]. The CRISPR phenotype scores for TgAP2XII-9 (TGME49_251740) and TgAP2III-2 (TGME49_253380) were -4.32 and -3.22, respectively, suggesting that both genes likely contribute to the parasite’s fitness. To elucidate their role in regulating the cell cycle expression program of tachyzoites, we used an auxin-inducible degron (AID) system. This involved fusing the AID sequence and an HA-tag to the C-terminus of TgAP2XII-9 and TgAP2III-2 at their respective endogenous locus via a CRISPR/Cas9 strategy (S1A Fig). PCR confirmed the correct integration of the tag (S1Bi and S1Bii Fig). The AID system allows conditional depletion of the protein upon adding Auxin to the parasite’s growth medium. Western Blot analysis validated the system’s functionality, showing a distinct band at the expected protein’s size (218 kDa and 185 kDa) in the absence of auxin (Fig 1A and 1B), with protein depletion occurring within 30 minutes of auxin addition for iKD TgAP2XII-9 and about 1 hour for iKD TgAP2III-2.

Subsequently, immunofluorescence assays using cell cycle markers such as TgCentrin1 (marking the outer core of the centrosome) revealed that TgAP2XII-9 and TgAP2III-2 localize to the nucleus and are expressed during the late S and early M phase (Fig 1C and 1D). Their expression is absent during the G1 phase before centrosome duplication and early S phase when centrosomes have duplicated but remain in close proximity to each other (Fig 1C and 1D, first two panels). During the late S and M phases, when centrosomes are segregated and when daughter cell formation (indicated by the budding marker TgIMC1) occurs, TgAP2XII-9 and TgAP2III-2 proteins are expressed (Fig 1C and 1D, last two panels), indicating that their expression peaks after that of TgAP2IX-5. Previous studies by Behnke et al. (2010) [30] and Khelifa et al. (2021) [27] revealed that TgAP2IX-5 expression peaks earlier in the cell cycle. TgAP2IX-5 controls the expression of TgAP2XII-9 and TgAP2III-2, which is consistent with their sequential roles in coordinating cell cycle progression and daughter cell formation.

### TgAP2XII-9 is crucial for the growth and proliferation of tachyzoites in vitro

To functionally characterize TgAP2XII-9 and TgAP2III-2, we performed a standard growth assay to assess parasite replication within host cells 24 hours after adding auxin. We found that for TgAP2XII-9, the mean number of parasites per vacuole decreased to 2.8, while in the controls, the mean number was ~4.4 (Fig 1E). This indicates a clear defect in parasite proliferation. Further phenotypic analyses revealed that the degradation of TgAP2XII-9 significantly impairs replication capacity *in vitro*. In a plaque assay, mutant parasites exposed to auxin failed to form any lysis plaques on a monolayer of host cells 7 days post-infection, unlike the parental strain under the same conditions (Fig 1F). Quantifying plaque numbers in each strain (Fig 1G), with and without auxin, also demonstrates that TgAP2XII-9 expression is crucial for the parasite’s growth and proliferation *in vitro*. In contrast, the TgAP2III-2 iKD strain did not show any replication defects in the presence or absence of auxin and plaque assays showed a normal capacity to form plaques in the presence of auxin and after TgAP2III-2 depletion (S1Ci and S1Cii Fig). This indicates that TgAP2XII-9 is crucial for tachyzoite proliferation while TgAP2III-2 is dispensable.

### TgAP2XII-9 is crucial for the proper formation of daughter parasites

**Fig 2:**
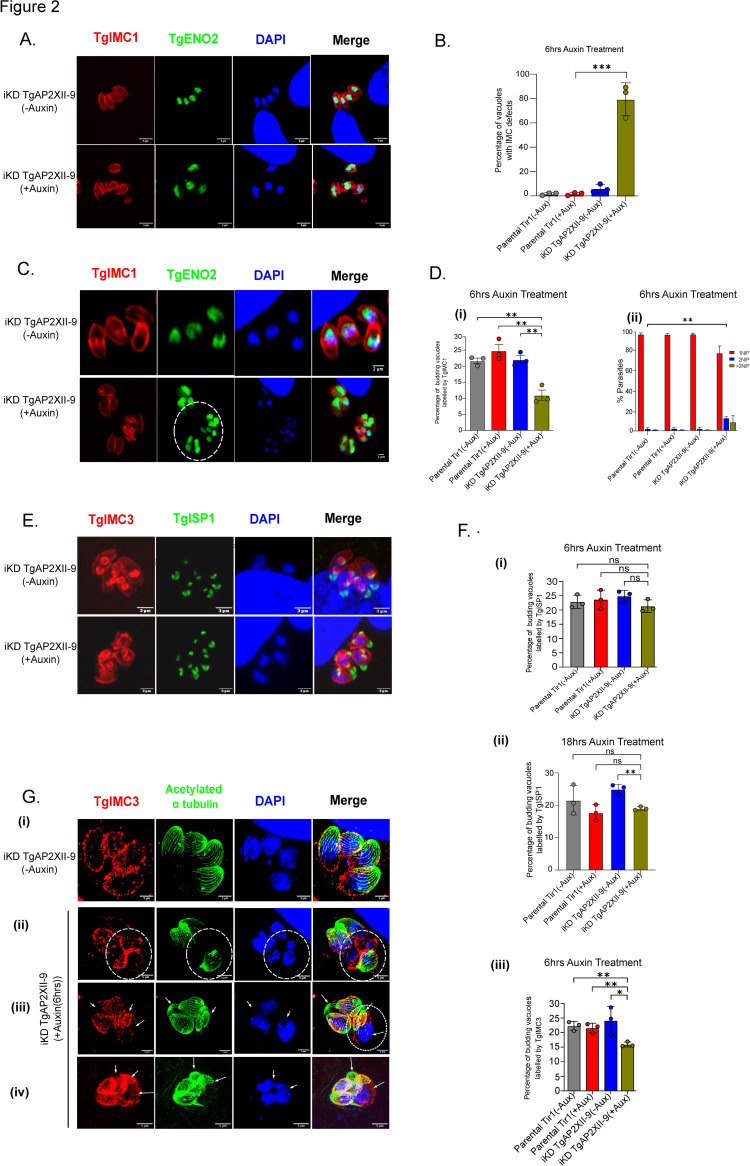
TgAP2XII-9 is crucial for the proper formation of daughter parasites. **(A)** Defects in the IMC formation or organization observed after 6 hours Auxin treatment as realized by IFA and confocal imaging with TgIMC1 and TgENO2; Scale bar = 5μm **(B)** Quantification of the IMC defect phenotype between the parental and iKD TgAP2XII-9 strains on 6hrs of auxin treatment. Statistical analysis was performed using a two-tailed Student’s t-test, with significance indicated by ***p < 0.001, data are presented as mean ± s.d. (n  =  3) **(C)** IFA and confocal imaging depicting the budding of iKD TgAP2XII-9 parasites and accumulation of multiple nuclei after the addition of Auxin using anti-TgIMC1 and anti-TgENO2 antibodies; scale bar = 2μm **(D)(i)** Quantification of percentage of vacuoles undergoing budding after 6hrs auxin treatment. Statistical analysis was performed using a two-tailed Student’s t-test, with significance indicated by **p < 0.01, data are presented as mean ± s.d. (n  =  3). **(D)(ii)** Quantification of percentage of vacuoles having multiple nuclei. Statistical analysis was performed using a two-tailed Student’s t-test, with significance indicated by **p < 0.01, data are presented as mean ± s.d. (n  =  3), N represents nuclei and P is parasites. **(E)** IFA and confocal imaging depicting the budding of parasites labelled by TgIMC3 and TgISP1; scale bar = 3 μm. **(F) (i)** Quantification of budding vacuoles using anti-TgISP1 after 6hrs of Auxin treatment, a two tailed Student’s Statistical analysis was performed using a two-tailed Student’s T-test, with significance indicated by ns>0.05, data are presented as mean ± s.d. (n  =  3). **(F) (ii)** Quantification of budding vacuoles using anti-TgISP1 after 18hrs of Auxin treatment, a two tailed Student’s Statistical analysis was performed using a two-tailed Student’s t-test, with significance indicated by ns>0.05, **p < 0.01 data are presented as mean ± s.d. (n  =  3). **(F) (iii)** Quantification of budding vacuoles using anti-TgIMC3 after 6hrs of Auxin treatment, Statistical analysis was performed using a two-tailed Student’s t-test, with significance indicated by **p < 0.01, *p<0.05, data are presented as mean ± s.d. (n  =  3). **(G)** Expansion microscopy depicting defects in the IMC (circled) and deformations in the cytoskeleton (panel (iv)). The parasite IMC (IMC3, red) and cytoskeleton (acetylated tubulin, green) were labelled as well as the nucleus by DAPI (blue). Parasite without nucleus(panel(iii)) and a parasite bearing multiple nuclei(panel(iii)) are indicated by white arrows. Scale bar = 5 μm.

To further explore the biological function of TgAP2XII-9, we conducted immunofluorescence assays (IFAs) to inspect the IMC formation (Fig 2A) after a short 6-hour auxin treatment (representing the time needed for the parasite to complete one cell cycle). In the absence of TgAP2XII-9, the parasites displayed defects in IMC scaffold organization, resulting in the formation of disordered IMCs (Fig 2A, lower panel). Conversely, TgAP2XII-9 parasites formed well-structured vacuoles without auxin (Fig 2A, upper panel). Quantitative analysis of this phenotype revealed that approximately 80% of vacuoles contained disorganized IMCs after 6 hours of auxin treatment (Fig 2B). Given the crucial role of the IMC in daughter cell formation, proper cellular content segregation, and daughter cell scaffold formation, we aimed to investigate the impact of TgAP2XII-9 on daughter cell development. Using TgIMC1 as a marker, we assessed the proportion of vacuoles undergoing budding following a 6-hour auxin exposure. It became evident that there was a notable decrease in the percentage of budding vacuoles, indicating the parasites’ compromised ability to generate daughter cells correctly compared to mutant parasites cultured without auxin (see Fig 2C and 2Di).

Additionally, the number of nuclei per parasite increased as a consequence of the inability of the daughter parasites to correctly bud, as depicted in Fig 2C (lower panel, iKD TgAP2XII-9 + Auxin, within the enclosed circle). Quantitative analysis revealed a significant proportion of vacuoles with two or more nuclei per parasite in the presence of auxin (Fig 2Dii), while this phenotype was nearly absent in the parental strain and the mutant strain in the absence of auxin. Taken together, these results suggest that TgAP2XII-9 is crucial for the proper formation and completion of the daughter cell scaffold.

### TgAP2XII-9 exhibits no discernible effect on the initiation of daughter parasite budding

To investigate the role of TgAP2XII-9 in the budding process, we used an early budding marker (TgISP1, apical cap) (Fig 2E) as a proxy for the budding initiation. Quantification of budding vacuoles after 6 hours of auxin treatment revealed no significant difference in the proportion of vacuoles undergoing budding (Fig 2Fi). However, after overnight (~18 hours) auxin treatment, there was a slight decrease in budding vacuoles compared to mutant parasites without auxin (Fig 2Fii). This reduction could be due to prolonged auxin exposure causing indirect effects rather than a direct result of TgAP2XII-9 depletion. Additionally, since TgIMC3 is strongly enriched in the daughter buds and is a later marker than TgISP1, we quantified budding parasites using TgIMC3. We observed a significant decrease in budding vacuoles after 6 hours of auxin treatment compared to parental and mutant strains without auxin (Fig 2Fiii) indicating that TgAP2XII-9 depletion does not affect budding initiation but rather elongation of IMC as early as the appearance of TgIMC3 on daughter buds.

To better visualize the defects in the daughter cell formation, we performed expansion microscopy (Fig 2G). Although the parasite cytoskeleton is formed (as marked by acetylated-α-Tubulin), we confirmed the deformation of the IMC (Circled, Fig 2Gii). As expected, we could identify parasites with an accumulation of multiple nuclei (Circled, Fig 2Gii). We also noticed that there were daughter parasites that were formed without a nucleus (circled, Fig 2Giii) and parasites that had no IMC or nucleus but with a well-formed cytoskeleton (arrowed, Fig 2Giii). There were also vacuoles with unorganized IMC and unsegregated nuclear material and abnormally formed cytoskeleton (arrowed, Fig 2Giv). These data indicate that TgAP2XII-9 has a profound influence on daughter cell formation and the coordination of this process.

We also examined the effect of TgAP2XII-9 depletion on organelle duplication and segregation. The Golgi complex and plastid were labelled in parasites (S2A Fig), and the Golgi-to-nucleus and plastid-to-nucleus ratios were calculated (S2Bi and S2Bii Fig). We found no significant difference in these ratios between the presence and absence of auxin, as expected since TgAP2XII-9 expression peaks after both organelle divisions. Overall, these findings suggest that TgAP2XII-9 is not involved in initiating daughter parasite budding but exerts its effects later in the budding cycle.

### TgAP2XII-9 and TgAP2III-2 have no combinatorial effect on the parasite biology *in vitro*

To explore the combinatorial effects of TgAP2XII-9 and TgAP2III-2 depletion, we generated a double mutant strain by knocking out TgAP2III-2 in our inducible knockdown (iKD) TgAP2XII-9 mutant strain. In this strain, in the absence of auxin, only TgAP2III-2 is depleted, while in the presence of auxin, both TgAP2XII-9 and TgAP2III-2 proteins are depleted. This strain is hereafter referred to as the Double Mutant. As anticipated, no plaques were observed in the Double Mutant strain in the presence of auxin (S3Ai and S3Aii Fig), consistent with the essential nature of TgAP2XII-9 for the parasite.

Quantitative growth assays revealed no additive impact on parasite proliferation due to the simultaneous depletion of TgAP2III-2 and TgAP2XII-9 (S3B Fig). Moreover, the presence of auxin led to the accumulation of multiple nuclei in the Double Mutant, mirroring the phenotype observed in the iKD TgAP2XII-9 strain (S3C and S3D Fig). Further examination of the IMC defect phenotype, for which TgAP2XII-9 is critical, indicated no additional defects in the Double Mutant compared to the iKD TgAP2XII-9 strain (S3C Fig). This was confirmed by the quantification of the percentage of vacuoles with IMC defects (S3E Fig) which remained similar to that of iKD TgAP2XII-9 single mutant. These findings collectively indicate that depleting both TgAP2III-2 and TgAP2XII-9 does not result in an additive effect on parasite biology.

### TgAP2XII-9 regulates the expression of various cell cycle-regulated genes

**Fig 3:**
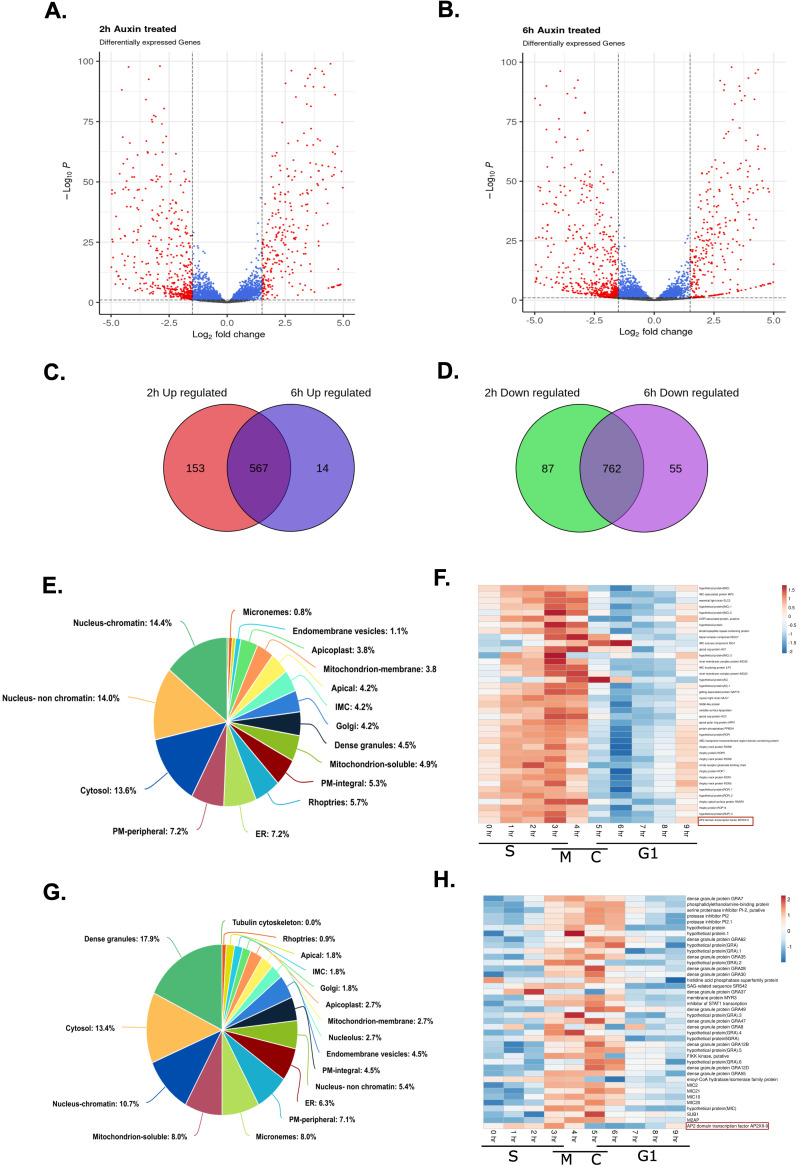
TgAP2XII-9 regulates the expression of various cell cycle-regulated genes. **(A)** Volcano plot of differentially expressed genes from RNA-sequencing analysis of TgAP2XII-9 parasites treated with auxin for 2 hours. **(B)** Volcano plot of differentially expressed genes from RNA-sequencing analysis of TgAP2XII-9 parasites treated with auxin for 6 hours. **(C)** Venn diagram showing the overlap of upregulated genes from 2h and 6h RNA-Seq data. **(D)** Venn diagram showing the overlap of downregulated genes from 2h and 6h RNA-Seq data. **(E)** Pie chart showing the percentage and localisation of upregulated transcripts from RNA-Seq data. **(F)** Heatmap showing the cell cycle expression of Rhoptry, IMC and Apical complex proteins encoding upregulated transcripts peaking simultaneously with TgAP2XII-9 expression (denoted in a box). The phases of the cell cycle are depicted at the bottom. **(G)** Pie chart showing the percentage and localisation of downregulated transcripts from RNA-Seq data. **(H)** Heatmap showing the cell cycle expression of micronemes and Dense granule proteins encoding downregulated transcripts. Expression of TgAP2XII-9 is denoted. The phases of the cell cycle are depicted at the bottom.

Given the potential role of TgAP2XII-9 as a transcription factor, we aimed to identify its regulated genes using RNA sequencing analysis. RNA sequencing was conducted at two time points: 2 hours post TgAP2XII-9 depletion to capture the genes immediately affected, and 6 hours post-depletion, corresponding to the completion of one tachyzoite cell cycle. Data analysis was performed using DESeq2 with an adjusted p-value cutoff of 0.01 and a minimum fold change of 2 (Fig 3A and 3B). Significant transcriptomic changes were observed in the iKD TgAP2XII-9 mutant, revealing 1569 (S1 Table) and 1398 (S2 Table) differentially expressed genes (DEGs) after 2 and 6 hours of auxin treatment, respectively. A substantial number of genes were common between both datasets. Overlapping these datasets resulted in a final set of 1329 DEGs, with 567 transcripts upregulated (Fig 3C and S3 Table) and 762 transcripts downregulated (Fig 3D and S4 Table) following TgAP2XII-9 depletion. We analysed the cell cycle expression of the upregulated and downregulated genes, displaying their expression profiles using a heatmap (S4A and S4B Fig). Majority of genes that were upregulated showed a peak expression during the late C and G1 phases, with a few peaking during the S phase (S4A Fig). The downregulated genes exhibited a heterogeneous expression pattern throughout the cell cycle, with peaks during the late S, M, and cytokinesis phases (S3B Fig).

Upon analyzing the differential enrichment between upregulated and downregulated transcripts encoding proteins localized to specific organelles in the parasites using the HyperLOPIT dataset from Barylyuk et al (2020) [31], we identified a notable trend: a significant proportion of the upregulated transcripts encode proteins that localize to the Rhoptries (5.7% of upregulated transcripts in the HyperLOPIT dataset; Fig 3E) compared to 0.9% of downregulated transcripts (Fig 3E and 3G), the apical compartment (4.2% of upregulated transcripts in the HyperLOPIT dataset; Fig 3E) compared to 1.8% of downregulated transcripts (Fig 3G), and the IMCs (4.2% of upregulated transcripts in the HyperLOPIT dataset; Fig 3E) compared to 1.8% of downregulated transcripts (Fig 3G). Additionally, we observed that a significant proportion of downregulated transcripts encode proteins that localize to the dense granules (17.9% of downregulated transcripts in the HyperLOPIT dataset; Fig 3G) compared to 4.5% of upregulated transcripts, and to the micronemes (8% of downregulated transcripts in the HyperLOPIT dataset; Fig 3G) compared to 0.8% of upregulated transcripts. According to the HyperLOPIT dataset, the upregulated genes comprise 17% of the apical proteome (11/63), 14% of the rhoptry proteome (15/106), and 14% of the IMC proteome (11/81). In contrast, the downregulated genes represent 15% of the dense granule proteome (19/124) and 18% of the microneme proteome (9/51).

Examining the cell cycle expression of the upregulated transcripts associated with Rhoptries, Apical Caps, and IMCs (where data is available) revealed that most of these transcripts peak concurrently with TgAP2XII-9 expression (Fig 3F). Conversely, the cell cycle expression of most of the downregulated transcripts associated with dense granules and micronemes peaked immediately after the expression of TgAP2XII-9 (Fig 3H).

### TgAP2XII-9 is enriched at the promoters of *T*. *gondii genes*

**Fig 4:**
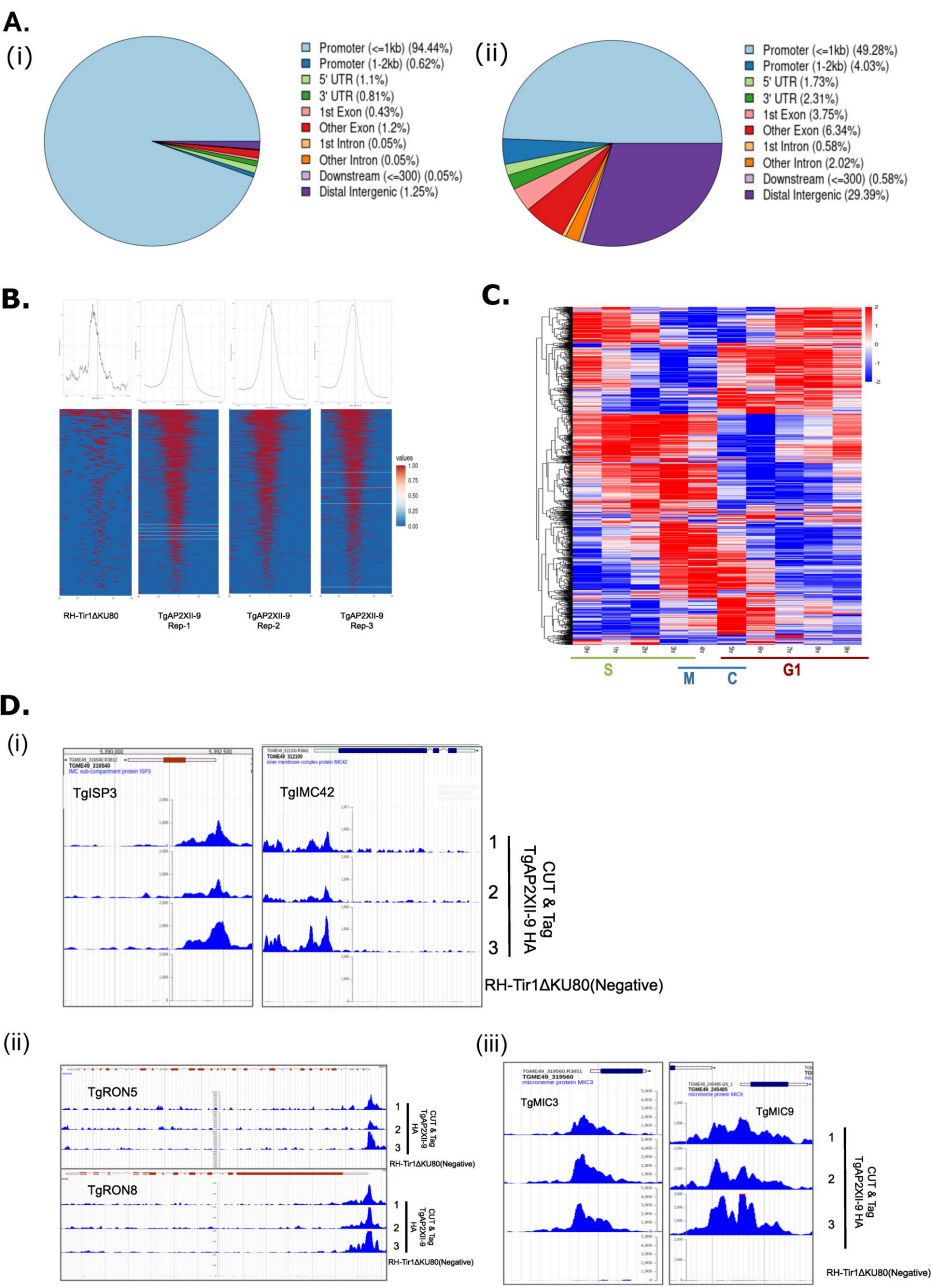
TgAP2XII-9 is enriched at the promoters of *T*. *gondii genes*. **(A) (i)** Pie chart showing the percentage of peaks (from 3 replicates) annotated in the different regions in the iKD TgAP2XII-9-HA strain. **(A) (ii)** Pie chart showing the percentage of peaks annotated in the different regions in the Parental Tir1 strain. (B) Density graphs and Heatmaps of the parental and TgAP2XII-9-HA (3 replicates) peaks located -2kb and +2kb from TSS. **(C)** Heatmap of cell cycle expression of all the genes that are bound by TgAP2XII-9 at their promoter. **(D)** CUT & Tag representing the direct targeting of TgAP2XII-9 to the promoters of TgISP3, TgIMC42 **(i)**, TgRON5, TgRON8**(ii)**, TgMIC3, TgMIC9**(iii).** Peak tracks for the 3 replicates of TgAP2XII-9-HA along with parental Tir1 are shown.

Using RNA-seq, we identified that TgAP2XII-9 regulates gene expression either directly or indirectly. To pinpoint the specific promoters targeted by TgAP2XII-9, we conducted CUT & Tag analysis. Biological triplicates of endogenous HA-tagged TgAP2XII-9 were generated, along with a replicate of the RH-Tir1ΔKU80 strain, and subjected to sequencing. Significant peaks (p-value < 0.05) were identified using MACS2 software. Subsequently, ChIPSeeker annotated these peaks, revealing that 95% were located at promoter sites for TgAP2XII-9 (Fig 4Ai) and 49% for RH-Tir1ΔKU80 (Fig 4Aii). Across the three replicates, 2088 (S5 Table) peaks were exclusive to TgAP2XII-9 and absent in RH-Tir1ΔKU80. In line with its hypothesized function as a transcriptional regulator, TgAP2XII-9 is found proximal to the transcription start sites of protein-coding genes (Fig 4B).

Analysis of cell cycle expression patterns revealed that genes with TgAP2XII-9-bound promoters predominantly exhibited peak expression during S and M phases (Fig 4D) in line with its expression pattern. CUT & Tag data demonstrated that TgAP2XII-9 binds to promoters of key genes encoding proteins crucial of the IMC, apical complex, rhoptries, micronemes, and dense granules (Fig 4D). Specifically, promoters of genes bound by TgAP2XII-9 include genes encoding for IMC proteins (e.g., TgISP3, TgIMC42; Fig 4Di), ROP proteins (e.g., TgRON5, TgRON8; Fig 4Dii), and MIC proteins (e.g., TgMIC9, TgMIC3; Fig 4Diii). These findings underscore TgAP2XII-9’s role as a genuine TF by directly interacting with promoters of genes essential for daughter cell formation.

**Fig 5:**
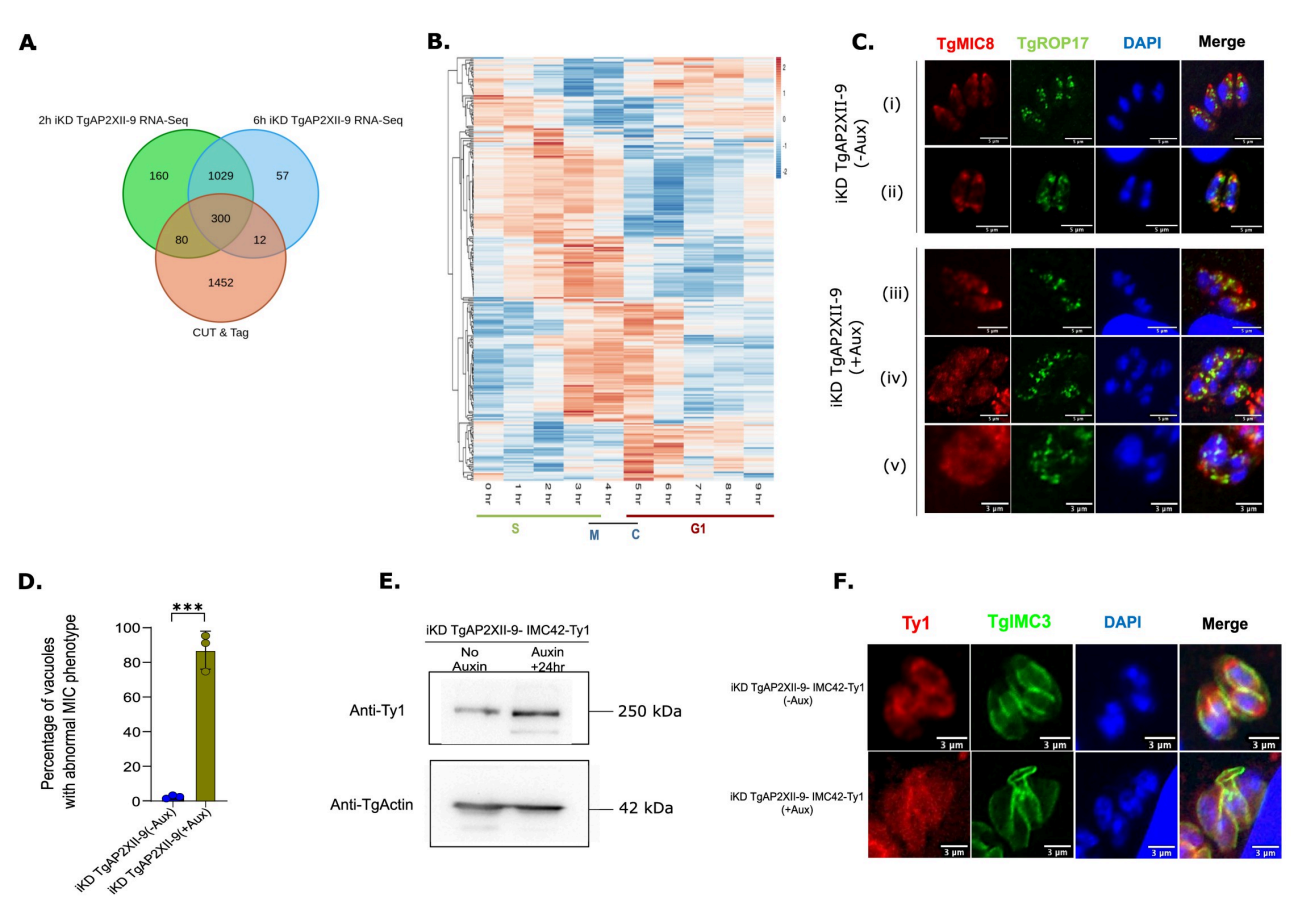
TgAP2XII-9 acts as a dual regulator regulating subset of MIC, ROP,GRA and IMC genes. **(A)** Venn diagram showing the overlap of genes from 2h RNA-Seq, 6h RNA-Seq and CUT & Tag data. **(B)** Heatmap of cell cycle expression of genes directly regulated and targeted by TgAP2XII-9. **(C)** Defects in the MIC organization observed after 24 hours Auxin treatment while ROPs remain unaffected as realized by IFA and confocal imaging with TgMIC8 and TgROP17; Scale bar indicated at the bottom right corner of each image **(D)** Quantification of the MIC abnormal phenotype between the parental and iKD TgAP2XII-9 strains on 24hrs of auxin treatment. Statistical analysis was performed using a two-tailed Student’s t-test, with significance indicated by ***p < 0.001, data are presented as mean ± s.d. (n  =  3) **(E)** Western blot analysis of total protein extracts from iKD TgAP2XII-9-IMC42-Ty1 strain treated with Auxin for 24hrs. The blots were probed with anti-Ty1 to detect TgIMC42 protein levels (upper panel) and with anti-TgSortilin as a normalization control (lower panel). **(F)** Defects in the IMC42 organization observed after 24 hours Auxin treatment as realized by IFA and confocal imaging with Ty1 and TgIMC3; Scale bar indicated at the bottom right corner of each image.

Since RNA-seq alone cannot identify genes directly regulated by TgAP2XII-9, we integrated RNA-seq with CUT & Tag datasets. We identified differentially expressed genes (both upregulated and downregulated) from the RNA-seq data and compared them with genes targeted by TgAP2XII-9 based on CUT & Tag analysis. This comparison revealed an overlap of 300 genes (Fig 5A and S6 Table). Detailed analysis showed that 31% of the upregulated genes and 16% of the downregulated genes are directly targeted by TgAP2XII-9, indicating that TgAP2XII-9 regulates genes mostly expressed during the S and M phases and some expressed during the cytokinesis phase (Fig 5B). Our results reveal that TgAP2XII-9 serves as a versatile transcriptional regulator with dual functions: it represses specific rhoptry and IMC genes while activating a subset of microneme and dense granule genes. In the light of these results, we wanted to verify if TgAP2XII-9 depletion had a direct effect on rhoptry or micronemes biogenesis. Therefore, we performed IFA using TgMIC8 (microneme marker) and TgROP17 (rhoptry marker) (Fig 5C). Upon auxin-induced depletion of TgAP2XII-9, we observed a significant disruption in microneme formation while rhoptry formation was unaffected. Specifically, in Fig 5Ciii, the micronemes are absent in budding daughter cells, although rhoptries appear unaffected. Furthermore, in Fig 5Civ, parasites with multiple nuclei are observed without a corresponding increase in micronemes, indicating a defect in organelle formation. Additionally, Fig 5Cv shows distorted and improperly localized microneme signals. Quantification of vacuoles with abnormal microneme signals upon TgAP2XII-9 depletion (Fig 5D) confirmed a statistically significant increase in abnormal localization patterns, supporting its function in microneme biogenesis.

Conversely, TgAP2XII-9 appears to act as a repressor of certain IMC genes. To confirm this, we investigated TgIMC42, whose promoter is bound by TgAP2XII-9 and is upregulated upon depletion of the transcription factor. In an iKD TgAP2XII-9 strain with Ty-tagged TgIMC42, Western blot analysis showed an increase in TgIMC42 protein levels following auxin treatment (Fig 5E), supporting the repressive role of TgAP2XII-9 on TgIMC42 expression. IFA of TgIMC42-Ty1 (red) and TgIMC3 (green) further confirmed TgAP2XII-9’s role in maintaining IMC organization (Fig 5F).

These findings collectively support a model in which TgAP2XII-9 functions as an activator for microneme genes leading to the proper microneme formation. Conversely, it represses certain rhoptry, IMC, and apical genes, whose expression peaks earlier in the cycle than TgAP2XII-9.

### Interplay of ApiAP2 transcription factors in regulating cell cycle and differentiation in *T*. *gondii*

**Fig 6:**
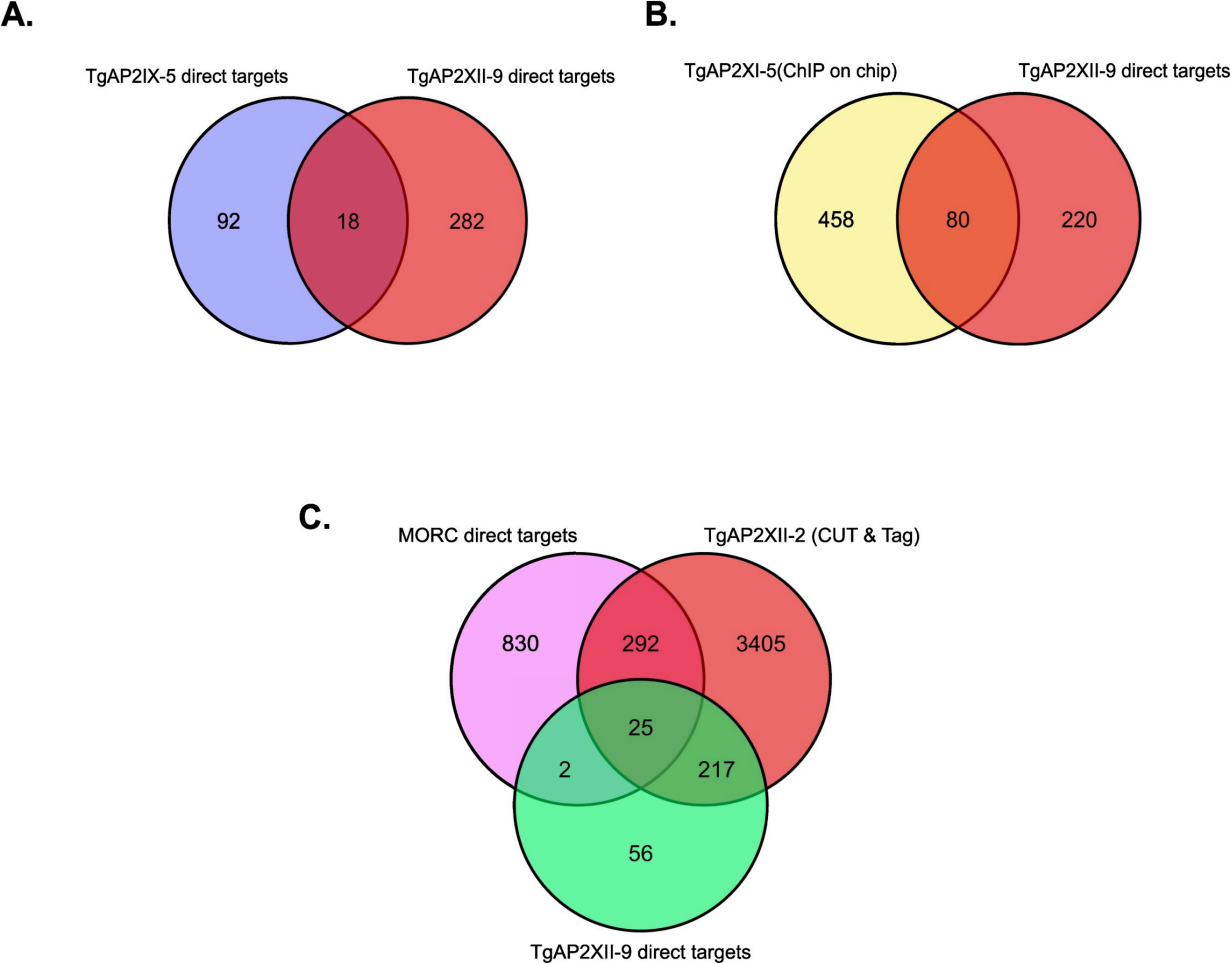
TgAP2XII-9 directly regulates key genes involved in the daughter parasite formation. **(A)** Venn diagram indicating that majority of genes targeted by TgAP2XII-9 are different to that targeted by TgAP2IX-5. **(B)** Venn diagram indicating that majority of genes targeted by TgAP2XII-9 are different to that targeted by TgAP2XI-5. **(C)** Venn diagram indicating the overlap of genes bound by both TgAP2XII-9 and TgAP2XII-2 and direct targets of MORC.

Given TgAP2IX-5’s role in activating genes involved in daughter parasite formation, we examined genes regulated by both TgAP2XII-9 and TgAP2IX-5. Out of the 300 genes directly targeted and regulated by TgAP2XII-9, only 18 were also targeted by TgAP2IX-5 (Fig 6A). Nearly all these genes are initially activated by TgAP2IX-5 and later repressed by TgAP2XII-9, including TgAP2XII-9 itself. These findings suggest that TgAP2XII-9 regulates a distinct set of genes and acts downstream of TgAP2IX-5.

We also looked at genes that are coregulated by TgAP2XI-5 and TgAP2XII-9. We overlapped the ChIP-on-chip data of TgAP2XI-5 [32] and identified 80 genes that were also bound by TgAP2XI-5 at their promoters (Fig 6B), in line with previous results showing that TgAP2XI-5 binds to promoter of genes preferentially expressed during the S and M phase.

Since TgAP2XII-9 and TgAP2XII-2 share a similar transcriptomic profile and are activated by TgAP2IX-5, we investigated whether TgAP2XII-2 also targets the direct targets of TgAP2XII-9. To this end, we compared our data with the CUT&Tag data of TgAP2XII-2 [33]. Remarkably, out of the 300 direct targets of TgAP2XII-9, 242 were also bound by TgAP2XII-2 at their promoters (Fig 6C). However, the expression of most of these genes did not vary after TgAP2XII-2 depletion [33]. Given that TgAP2XII-2 is known to interact with the TgMORC/TgHDAC3 complex [33], we examined whether these 242 genes are also targets of TgMORC. We compared this data with the ChIP-seq data of MORC [34]. Only 25 genes were bound by TgMORC at their promoters (Fig 6C), and these genes did not belong to any specific gene set. Notably, two AP2 transcription factors (TgAP2Ib-1 and TgAP2IV-3), involved in bradyzoite-specific gene expression, were present. This data indicate that TgAP2XII-2 has a different biological function than TgAP2XII-9 although they bind to similar promoters.When examining the 25 genes whose promoter is bound by TgAP2XII-2, TgAP2XII-9 and TgMORC, we found that most of them had a peak expression during the M and Cytokinesis phases (S5A Fig). All these 25 genes were highly expressed throughout the sexual stages with their expression peaking at EES5 and tissue cysts (S5B Fig).

**Fig 7:**
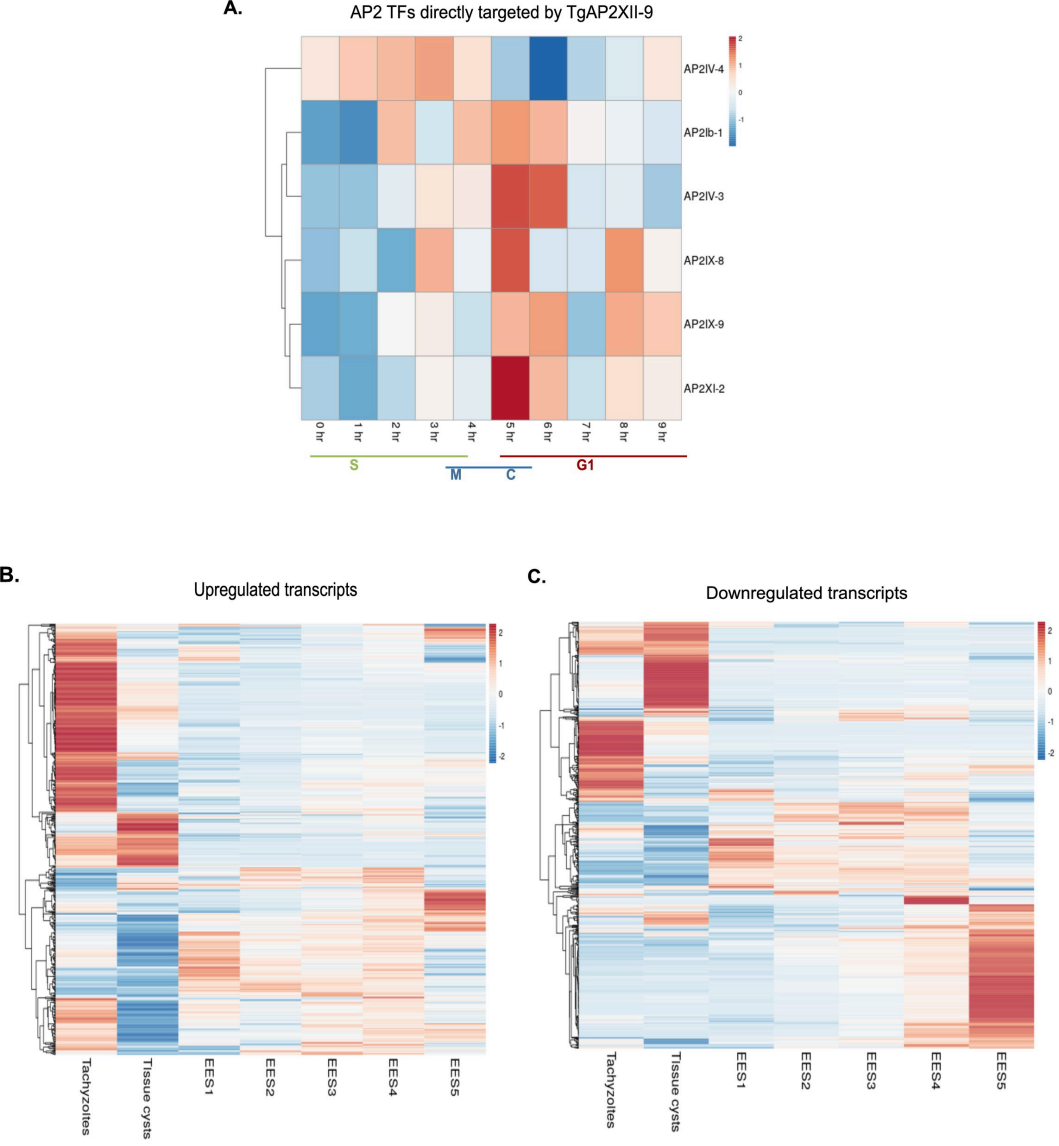
TgAP2XII-9 directly regulates other AP2 transcription factors and might provide a conducive environment for the expression of genes preferentially expressed in bradyzoites. **(A)** Heatmap of cell cycle expression of the AP2 TFs directly regulated by TgAP2XII-9**. (B)** Heatmap of expression of upregulated transcripts through the tachyzoite, bradyzoite and sexual stages. Most of the overexpressed transcripts upon TgAP2XII-9 depletion are preferentially expressed at the tachyzoite stage of the parasite **(C)** Heatmap of expression of downregulated transcripts through the tachyzoite, bradyzoite and sexual stages. Many of the downregulated transcripts upon TgAP2XII-9 depletion are preferentially expressed at bradyzoite and throughout the sexual stages especially during EES5. EES stands for enteroepithelial developmental stages. Source data for B&C–Ramakrishnan et al.,2019 [35].

We also investigated other ApiAP2 TFs potentially regulated by TgAP2XII-9 and found that 7 ApiAP2 TFs were bound and regulated by TgAP2XII-9. Of these, four AP2s (TgAP2IV-4, TgAP2IX-8, TgAP2IX-9, TgAP2XI-2) were upregulated when TgAP2XII-9 was depleted, and 2 AP2s (TgAP2IV-3, TgAP2Ib-1) were downregulated. Analysis of the cell cycle expression of these AP2s suggests that most peak during the late C and early G1 phases, except for TgAP2IV-4, which peaks during the S/M phase along with TgAP2XII-9 (Fig 7A). To investigate the link between TgAP2XII-9 and differentiation, we examined the expression profile of upregulated and downregulated genes during the parasite life cycle (Fig 7B) using the data from Ramakrishnan *et al*. (2019) [35]. Interestingly, downregulated genes in the absence of TgAP2XII-9 are preferentially expressed in tachyzoite but also bradyzoites and sexual stages (Fig 7C), while TgAP2XII-9 depletion induced the overexpression of mostly tachyzoite-specific genes (Fig 7B). These data indicate that AP2XII-9 may produce a permissive environment for expression of genes that preferentially expressed in bradyzoites and sexual stages.

Surprisingly, TgAP2XII-9 was enriched at its own promoter (S6A Fig), and the TgAP2XII-9 transcript was upregulated in the presence of auxin based on RNA-seq. These data suggest that TgAP2XII-9 may directly regulate its own transcript expression, indicating a possible negative feedback loop.

### Complementation restores TgAP2XII-9 phenotypes observed

**Fig 8:**
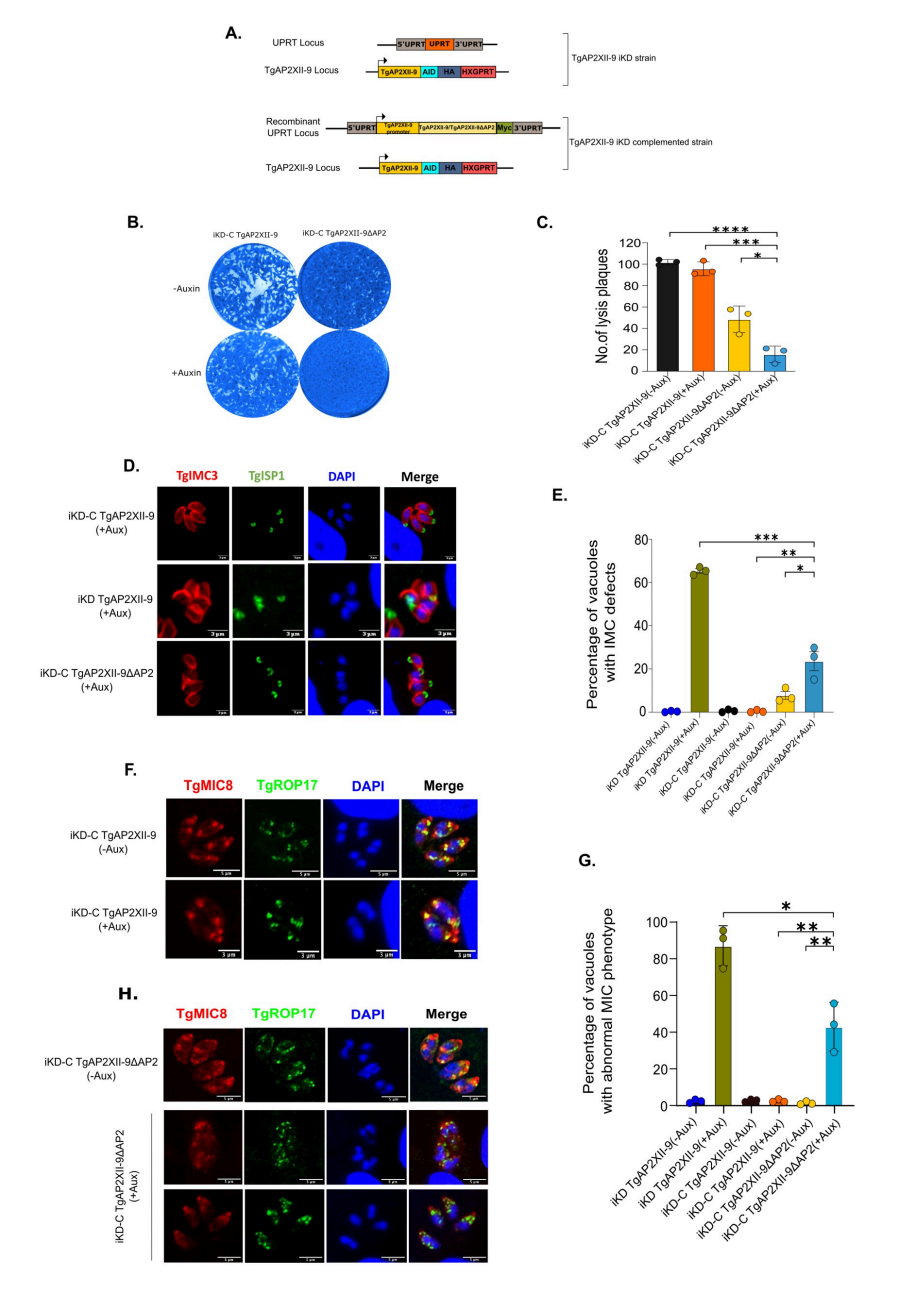
TgAP2XII-9 is responsible for the phenotypes observed and the AP2 domain is crucial for its function. **(A)** Schematic representation of the iKD TgAP2XII-9 complementation strategy. The UPRT locus was targeted for the insertion of exogenous myc-tagged TgAP2XII-9/TgAP2XII-9ΔAP2, driven by its native promoter, to generate the complemented TgAP2XII-9 iKD strain. **(B)** Plaque assay showing the proliferation of the iKD-C TgAP2XII-9 and iKD-C TgAP2XII-9ΔAP2 strains in the presence and the absence of Auxin. **(C)** Quantification of the number of plaques in the iKD-C TgAP2XII-9 and iKD-C TgAP2XII-9ΔAP2 strains. Statistical analysis was performed using a two-tailed Student’s t-test, with significance indicated by ****p<0.0001, ***p < 0.001, *p<0.05. Data are presented as mean ± s.d. (n  =  3). **(D)** IFA and confocal imaging illustrate the IMC defect phenotype seen by using TgIMC3 and TgISP1 in the iKD TgAP2XII-9 and iKD-C TgAP2XII-9ΔAP2 in presence of Auxin but not in the iKD-C TgAP2XII-9, scale bar = 3 μm**. (E)** Quantification of the IMC defect phenotype using TgIMC3 between iKD TgAP2XII-9, iKD-C TgAP2XII-9 and iKD-C TgAP2XII-9ΔAP2 strains. Statistical analysis was performed using a two-tailed Student’s t-test, with significance indicated by *p<0.05, **p<0.01, ***p < 0.001, Data are presented as mean ± s.d. (n  =  3).

We created a complemented strain (iKD-C TgAP2XII-9) by inserting a myc-tagged version of the TgAP2XII-9 gene, driven by its own promoter, into an exogenous locus (*uprt*; Fig 8A). The expression and localization of the exogenous TgAP2XII-9-myc in this strain were confirmed through IFA (S6B Fig). We compared the percentage of parasites expressing the myc-tagged copy with those expressing the endogenous HA-tagged version. About 30% of the asynchronous parasite population expressed the myc-tagged gene in the complemented strain, similar to the parental iKD TgAP2XII-9 strain (S6C Fig).

To assess if the iKD TgAP2XII-9 strain phenotype could be rescued by ectopic expression of TgAP2XII-9, we measured the percentage of vacuoles with IMC defects in the iKD-C TgAP2XII-9 strain both in the absence and presence of auxin (S6D Fig). We found very few to no vacuoles with IMC defects, indicating that the IMC defect phenotype observed in the iKD TgAP2XII-9 strain was due to the lack of TgAP2XII-9 protein. Furthermore, plaque assays showed that the parasites could form lysis plaques even in the presence of auxin, similar to their behaviour in the absence of auxin (Fig 8B). These results demonstrate that the TgAP2XII-9 protein is responsible for the phenotypes observed.

### The TgAP2XII-9 AP2 domain is crucial for its function

We investigated the role of the AP2 domain in TgAP2XII-9 function by modifying the complementation plasmid previously used to create the iKD-C TgAP2XII-9 strain, deleting the AP2 domain (Fig 8A). This complemented strain, designated iKD-C TgAP2XII-9 ΔAP2, was verified for expression and localization of the exogenous iKD-C TgAP2XII-9 ΔAP2 protein via immunofluorescence (S6E Fig).

To assess the impact of AP2 domain deletion, we conducted a plaque assay. HFF cell monolayers inoculated with iKD-C TgAP2XII-9 ΔAP2 parasites in the presence of auxin showed little to no lysis plaques, unlike the lysis plaques observed in wells infected with iKD-C TgAP2XII-9 parasites (Fig 8B and 8C). Interestingly, co-expression of the endogenous TgAP2XII-9 protein and the AP2-deleted exogenous protein had a deleterious effect on parasite growth, as seen by the reduced plaque formation in the iKD-C TgAP2XII-9 ΔAP2 strain even in the absence of auxin (Fig 8C).

We next examined the phenotype of abnormal IMC defects, recording the proportion of vacuoles exhibiting this feature (Fig 8D). Only a small percentage of vacuoles displayed this phenotype, suggesting that TgAP2XII-9 ΔAP2 could partially complement the IMC defect (Fig 8E). We also analyzed the microneme biogenesis phenotype across the iKD, complement, and ΔAP2 strains, quantifying the results (Fig 8F and 8H). Approximately 40% of vacuoles in the TgAP2XII-9 ΔAP2 strain exhibited an abnormal MIC phenotype in the presence of Auxin (Fig 8G). Overall, these findings indicate that the AP2 domain is crucial for TgAP2XII-9’s function, though it does not fully account for the phenotypes observed.

## Discussion

Proliferation is key to *T*. *gondii* pathogenesis in the intermediate hosts. The tachyzoite employs a unique, rapid replication method where daughter parasites are formed within a single mother cell (endodyogeny). However, how gene expression is regulated during this process is only partially understood.

Our study corroborates the findings of Shi et al. (2024) [36] regarding the essential role of TgAP2XII-9. However, since the data generated by Shi *et al*. [36] is produced after 24-hour auxin treatment, a direct comparison between the phenotypes observed in their study or RNA-seq data is not feasible. By focusing on short auxin treatments (2h or 6h), our approach aimed to identify the direct, early consequences of TgAP2XII-9 depletion. When we compared our CUT & Tag data with the corresponding dataset from Shi et al. [36], we found a significant overlap, with more than 50% of their identified targets also appearing in our data. This overlap reinforces the validity of our findings and underscores the critical role of TgAP2XII-9 in the parasite’s biology.

In our study, we have characterized two cell cycle-regulated ApiAP2 TFs (TgAP2XII-9 and TgAP2III-2) that are predominantly expressed during the S/M phase of the tachyzoite cell cycle. While TgAP2III-2 has no measurable impact on the ability of the tachyzoite to grow, TgAP2XII-9 depletion resulted in significant defects in daughter bud formation and disorganization of the IMC. Both TFs were shown to be regulated by TgAP2IX-5 [27] and we hypothesized that they may be important for the continuation of the cell cycle. TgAP2IX-5 is a crucial TF regulating the initiation of budding and we expected both TgAP2XII-9 and TgAP2III-2 to regulate subsequent steps of the cell cycle. Depletion of TgAP2III-2 did not cause a defect in parasite proliferation. In contrast, we show that TgAP2XII-9 is important for the formation of daughter cells but does not prevent the initiation of budding and the IMC apical cap formation (Fig 2). Instead, TgAP2XII-9 is important for IMC elongation and proper formation of the buds. Much like the erythrocytic cycle of *Plasmodium* [37] the identification of tachyzoite cell cycle-regulated expression profiles [30] suggests the presence of a cascade of TFs regulating this process.

This is the first evidence that the tachyzoite cell cycle-regulated expression program is controlled by a series of ApiAP2 TFs, cascading to implement the specific expression programs at each phase of the tachyzoite cell cycle. TgAP2IX-5 controls the expression of TgAP2XII-9 (also TgAP2XII-2 and TgAP2III-2) which in turn establishes the crucial expression profiles required for the progression of the cell cycle by coordinating the temporal expression of many transcripts.

TgAP2IX-5 controls genes that are essential for budding initiation, while TgAP2XII-9 seems important for the subsequent phases of daughter cell construction. When examining the genes that are directly controlled by TgAP2XII-9 (genes both present in the RNA-seq and CUT&Tag dataset), we noticed that genes encoding for TgISP3 and Apical Annuli proteins TgAAP5 and TgAAMT were downregulated. These proteins are expressed after the IMC apical cap deposition and are present at the central IMC sub compartment (TgISP3) or the apical annuli (TgAAP5 and TgAAMT). Interestingly, TgISP3 maternal staining dissipates as daughter parasites form, indicating that TgISP3 may be synthesized in daughters and degraded in mothers [38]. Our data confirm this hypothesis and suggest an active role of TgAP2XII-9 in this process. Apical annuli proteins may be inserted in the suture of the IMC plaques and therefore are needed once the apical and central IMC plaques are formed [39]. While TgAP2XII-9 seems to activate the expression of some IMC proteins, our analysis also detected IMC and basal complex genes that were directly repressed by TgAP2XII-9. Notably, the expression of transcripts encoding TgAC1 and TgIAP2, two apical cap proteins, and TgBCC3 and TgBCC7, two early and late markers of the basal complex [40–42] are directly repressed by TgAP2XII-9. These data indicate that TgAP2XII-9 seems to exert a dual activity of repressing the early budding markers (e.g apical cap and early basal complex component) and activating the expression of transcript encoding for proteins needed during IMC elongation. Overall, our data indicate that the production and assembly of each daughter cell IMC subcompartments correspond to a strictly controlled process that involves the timely expression of IMC encoded transcripts and proteins in different temporal waves that are controlled at least by TgAP2IX-5 and TgAP2XII-9. Indeed, it has been hypothesized that the apical cap of the IMC is assembled in the apical direction while the central and basal compartments are in the basal direction [43].

Defects in daughter cell formation have downstream effects on other phenotypes such as nuclear segregation. For example, TgILP1 overexpression, which predominantly localizes to budding daughters, leads to severely deformed cytoskeletons and abnormally large nuclei, suggesting a disruption in mitotic coordination similar to the phenotype that we see in TgAP2XII-9 depleted parasites [43]. Interestingly, *TgILP1* transcript is overexpressed in TgAP2XII-9 depleted parasites, recapitulating some of the phenotypes we observed. However, the IMC phenotype observed is probably due to the collective deregulation (up or downregulation upon TgAP2XII-9 depletion) of multiple genes targeted to the IMC and whose expression is controlled directly or indirectly by TgAP2XII-9.

Much like daughter cell IMC formation, the *de novo* production of rhoptries and micronemes are tightly regulated. Cell-cycle transcript expression profiles show that the temporal expression of rhoptries (early S phase) and micronemes (early M phase) are different [40]. Consistent with these gene expression patterns, we showed that transcripts encoding rhoptry proteins, which peak prior to TgAP2XII-9 expression are repressed by this TF, whereas expression of transcripts encoding microneme proteins is activated by TgAP2XII-9. We also showed that TgAP2XII-9 ensures the proper biogenesis of micronemes probably through the activation of the expression of micronemes genes. TgAP2XI-5 and TgAP2X-5 were already shown to be important in regulating the expression of virulence factor genes, specifically as activators of rhoptry-encoded transcripts [20]. Our findings indicate that transcript expression profiles linked to the cell cycle, similar to those observed during differentiation, must be kept repressed until their expression becomes necessary. This regulatory pattern is particularly evident for transcripts encoding rhoptry and microneme proteins, which need to be expressed precisely when these organelles are formed *de novo*. Thus, TgAP2XII-9 is positioned as a repressor of a subset of rhoptry protein encoded transcripts and an activator of a subset of microneme protein encoded transcripts, contrasting with the roles of TgAP2XI-5 and TgAP2X-5, which primarily activate transcripts encoding rhoptry proteins.

Upon TgAP2XII-9 depletion, some kinase transcripts were upregulated. Cyclin-related kinases like TgCRK1 and TgCRK6 are significantly dysregulated after TgAP2XII-9 depletion as suggested by our RNAseq data. Notably, the TgCRK6 promoter is also directly bound by TgAP2XII-9 and its transcript expression is being repressed. It is interesting to note that TgCRK6 interacts with TgAP2IX-5 and has similar temporal expression and localization to that of TgAP2IX-5 [44] It was also speculated that the role of TgCRK6 might be to inactivate TgAP2IX-5 just after budding has been initiated [44]. If in fact that is the case, TgAP2XII-9 (activated by AP2IX-5) might be repressing the gene expression of TgCrk6 when it is no longer needed, for example, after budding initiation. Overall, our data show that TgAP2XII-9 is acting as a crucial transcription factor at a turning point during the cell cycle when daughter cell buds are formed and microneme biogenesis must occur.

We identified that a majority of the genes that are downregulated in response to *TgAP2XII-9* knockdown are preferentially expressed in bradyzoites or sexual stages compared to tachyzoites (Fig 6C). This is probably linked to the list of ApiAP2 TFs that are dysregulated after TgAP2XII-9 depletion. Nine AP2 transcription factors are significantly upregulated (TgAP2IV-4, TgAP2IX-8, TgAP2XI-2, TgAP2IX-9, TgAP2XII-9, TgAP2IV-2, TgAP2XI-3, TgAP2XI-4 and TgAP2III-2) and 4 are significantly downregulated (TgAP2IV-3, TgAP2Ib-1, TgAP2IX-3 and TgAP2IV-1). Of these TgAP2XII-9 binds to the promoters of TgAP2IV-4, TgAP2IX-8, TgAP2XI-2, TgAP2IX-9, TgAP2XII-9, TgAP2IV-3, and TgAP2Ib-1. TgAP2IV-4 and TgAP2IX-9 are known repressors of bradyzoite-specific genes [45,46], whereas TgAP2IV-3, TgAP2Ib-1 and TgAP2XI-4 are activators [47,48]. These data suggest that TgAP2XII-9 directly represses the transcripts of other AP2s, such as TgAP2IV-4 and TgAP2IX-9, which in turn may repress bradyzoite-specific gene expression, while it activates the expression of AP2IV-3 and AP2Ib-1, which stimulate the bradyzoite-specific gene expression. This is reminiscent of the data published on TgAP2IX-5 [27], which was shown to activate the expression of TgAP2IV-4 (a repressor of bradyzoite differentiation). These data reinforce the link between the cell cycle and differentiation that was previously shown [49,50] where AP2 TFs important for differentiation are expressed at a specific point of the cell cycle (early M phase). At this specific point, by controlling the expression of a subset of AP2 TFs, TgAP2XII-9 might create a more permissive environment for the bradyzoite expression program and offer a possible getaway toward bradyzoite differentiation. In contrast, TgAP2IX-5, which acts to initiate budding, promotes the repression of the bradyzoite-specific expression program.

The complementation using the AP2 deleted copy of the TgAP2XII-9 gene was instrumental in showing the importance of the AP2 domain in TgAP2XII-9’s biological activity. However, the phenotypes could be partially complemented by this deleted copy of the genes indicating that TgAP2XII-9 may interact with other proteins to exert its action. We and other have previously shown that ApiAP2 protein can bind to DNA as hetero or homodimers, suggesting that the cooperative nature of their activity [20,51].

By the use of different complementation constructs, we examined the role of the AP2 domain in the function of TgAP2XII-9. As expected, the AP2 domain is critical for the essential function of TgAP2XII-9. However, when complementing the iKD strain using a construct deleted for the AP2 domain, some of the phenotypes, such as the disordered IMC phenotype, were partially complemented. This indicates that TgAP2XII-9 may exert some of this function through other parts of the proteins. ApiAP2 TFs are known to heterodimerize and cooperate to exert their function [20,51]. TgAP2XII-9 might therefore interact and cooperate with other proteins to regulate this phenotype independently from the presence of the AP2 domain.

Finally, we observed that TgAP2XII-9 binds to its own promoter and represses it which seems to be a typical characteristic of other AP2s. This feature was also shown for TgAP2IX-5 [27], TgAP2XI-5 [32], and TgAP2XII-2 [33]. This indicates that negative feedback loops are a common regulatory mechanism for these TFs and during the tachyzoite cell cycle, adding another layer of complexity to gene regulation in *T*. *gondii*.

In conclusion, we showed that TgAP2XII-9 plays a crucial role as a TF during daughter cell formation by activating genes that are required during the process of daughter cell IMC elongation and microneme *de novo* synthesis and repressing the expression of genes necessary during budding initiation.

## Materials & methods

### Parasite culture, transfection, and purification

The tachyzoites from the RH Δku80 Tir1 strain of *Toxoplasma gondii* were grown in human foreskin fibroblasts (HFF) under controlled laboratory conditions, using Dulbecco’s modified Eagles medium supplemented with 10% fetal calf serum (FCS), 2 mM glutamine, and 1% penicillin-streptomycin. This particular strain, RH Δku80 Tir1, is recognized for its rapid proliferation due to the deletion of the ku80 gene, which promotes successful homologous recombination and transfection. Moreover, this strain produces the Tir1 protein, facilitating the regulated breakdown of labelled proteins upon the introduction of auxin to the culture medium. The cultivation process occurred in ventilated tissue culture flasks within a HERA cell VLOS 160i CO2 incubator (Thermo Scientific) maintained at 37°C and 5% CO2. Transgenes were delivered through electroporation utilizing a BTX Harvard apparatus electroporator (ECM 630), and stable transformants were identified by growing them in media containing specific concentrations of mycophenolic acid (MPA)- 25 μg/ml, xanthine (50 μg/ml), pyrimethamine (2 μM), or FUDR (5 μg/ml). Clonal lines were isolated through a process of limited dilution. Before extracting total RNA, genomic DNA, or protein, intracellular parasites underwent purification via sequential syringe passage, first through a 17 gauge and then 26-gauge needles (Terumo AGANI needles) and filtration of the parasite through 3-μm polycarbonate membrane filter (Whatman).

### Generation of transgenic *T*. *gondii* strains

The iKD TgAP2XII-9 strain was developed by utilizing the RH Δku80 Tir1 strain, in conjunction with a Cas9 plasmid engineered to target the gene’s 3’ end post the stop codon, and a PCR product containing the HA-AID cassette flanked by homology regions. The primer sequences utilized in this experiment are detailed in the S7 Table. To produce the iKD and ΔAP2 complementation line, a plasmid containing 3-kb upstream of the predicted ATG of the TgAP2XII-9 gene and the full-length or AP2 domain-deleted c-myc-tagged TgAP2XII-9 gene flanked by 2 kb homology fragments for the uprt gene was co-transfected with the pSAG1::Cas9-U6::sgUPRT plasmid in the iKD TgAP2XII-9 strain to ensure insertion into the UPRT locus. The parasites were then selected using 5 μM 5-fluoro-2’-deoxyruridine (FUDR). To produce the double mutant strain, specifically a clean knockout of the TgAP2III-2 gene in the inducible knockdown (iKD) TgAP2XII-9 background, two gRNAs were designed to target the 5’ and 3’ ends of the TgAPIII-2 gene. This strategy facilitated the insertion of a DHFR selection cassette flanked by 35 base pair (bp) homology regions at both the 5’ and 3’ ends. The procedure involved transfecting the iKD TgAP2XII-9 parasite with two Cas9 plasmids, each targeting one end of the TgAPIII-2 gene.

### Growth assays

To assess growth, we introduced 8 x 10^4 parasites of both parental Tir1 and iKD AP2XII-9 mutant strains onto HFF cell monolayers cultivated on coverslips in a 24-well plate. This setup was maintained for 24 hours under conditions with and without 0.5mM auxin (AID/indoleacetic acid) in the medium. The purpose of incorporating auxin was to trigger the degradation of TgAP2XII-9 protein. Following 24-hour duration, infected coverslips were treated with 4% paraformaldehyde (PFA) for fixation. The fixed parasites were then subjected to staining using anti-TgEno2 to visualize parasite nuclei and anti-TgIMC1 antibodies to visualize the Inner Membrane Complex (IMC). The quantification involved counting the number of parasites per vacuole, with 100–200 vacuoles analyzed per biological replicate. Each growth assay experiment comprised three biological replicates.

### Plaque assay

Plaque assays were conducted by inoculating either 500 parasites of the Parental Tir1 strain or the iKD TgAP2XII-9/ TgAP2III-2 or the Double Mutant strain onto a monolayer of HFF cells cultivated in a 6-well plate, with the choice of normal media or media supplemented with 0.5mM auxin. The parasites were allowed to proliferate for 7 days before fixation with 100% ethanol. Plaques were visualized by staining with Crystal Violet. To assess plaque size under each experimental condition, an Excel macro was utilized for quantification.

### Organelle labelling

The Parental Tir1 and iKD TgAP2XII-9, TgAP2III-2 parasites were cultured on HFF cell monolayers on coverslips within 24-well plates. They were grown in both regular media and media supplemented with auxin for either overnight or 6 hours. Subsequently, the parasites were fixed using 4% PFA and stained with antibodies. The nucleus was marked using anti-TgEno2, and the count of nuclei per parasite was conducted. For the labeling of the Inner Membrane Complex, both the parental and iKD TgAP2XII-9 strains were allowed to grow on HFF cells for 18–20 hours, followed by a 6- or 18-hour treatment with auxin. Intracellular parasites were then labelled using anti-TgISP1 and anti-TgIMC1 antibodies. The components of the centrosome were labelled after overnight growth of both the parental and iKD TgAP2XII-9 strains, followed by a 6-hour auxin treatment, using anti-TgCentrin1 and anti-TgChromo1 antibodies. Golgi and plastid labelling were performed after overnight growth of both the parental and iKD TgAP2XII-9 strains in auxin-containing media, using anti-TgSortilin and anti-TgACP antibodies, respectively.

### Immunofluorescence assays (IFA)

Immunofluorescence experiments were conducted following the fixation of intracellular parasites cultivated on coverslips using 4% PFA for 30 minutes. Subsequently, the coverslips were washed three times with 1X PBS buffer. Permeabilization was achieved by incubating the samples for 30 minutes in a buffer composed of 1X PBS, 0.1% Triton 100X, 0.1% glycine, and 5% FBS. Following permeabilization, primary antibody incubation was performed for 1 hour, with the antibodies diluted in the same buffer used for permeabilization. Afterward, the coverslips containing the fixed intracellular parasites were washed three times with 1X PBS and incubated for 1 hour with DAPI and secondary antibodies conjugated to either Alexa-594 or Alexa-488. Following another three washes with 1X PBS buffer, the coverslips were mounted onto microscope slides using Moviol. Primary antibodies used included anti-TgIMC1 (a gift from Prof. Ward, University of Vermont), anti-TgEno2, anti-TgISP1, anti-TgCentrin1 (a gift from Prof. Gubbels, College of Boston), anti-TgACP (a gift from Pr. Striepen, U. Penn), anti-TgSortilin, and anti-HA (Sigma Aldrich), anti-myc (abcam), anti-TgIMC3(a gift from Prof. Gubbels, College of Boston) were used at the following dilutions: 1:500, 1:1000, 1:500, 1:500, 1:500, 1:500, 1:1000, 1:200 and 1:2000 respectively. Signal visualization involved manually counting 100–300 parasites for each replicate, with a total of three replicates carried out for each experiment. Immunofluorescence assay experiments were visualized using the ZEISS LSM880 confocal microscope at 63X magnification, and image processing was conducted using CARL Zeiss Zen software.

### Ultrastructure Expansion Microscopy (ExM) Procedure

Coverslips with HFF monolayers were inoculated with the iKD TgAP2XII-9 strain. The iKD strain was cultured either in normal media or media treated with auxin for 6 hours. Subsequently, cells were fixed with 4% paraformaldehyde (PFA) and prepared for ultrastructure expansion microscopy (U-ExM) following previously described protocol [52]. Briefly, the coverslips were incubated for 5 hours in a 2× 1.4% acrylamide (AA)/2% formaldehyde (FA) mix at 37°C. Gelation was performed by incubating in a solution containing ammonium persulfate (APS), tetramethylethylenediamine (TEMED), and a monomer mixture (19% sodium acrylate, 10% AA, and 0.1% bis-acrylamide in 10× PBS) for 1 hour at 37°C. The gels were then denatured at 95°C for 1.5 hours. Following denaturation, gels were incubated in double-distilled H2O (ddH2O) overnight to allow for expansion. The next day, gels were washed three times in PBS (10 minutes each) before incubation with primary antibodies for 3 hours at 37°C. After primary antibody incubation, gels were washed three times in PBS-Tween 0.1%, followed by incubation with secondary antibodies for 3 hours at 37°C. The gels were washed again three times in PBS-Tween 0.1% and then incubated in ddH2O for a second round of expansion before imaging. Confocal imaging was conducted using a ZEISS LSM880 Confocal Microscope at 63x magnification. Primary antibodies used were anti-TgIMC3 (a gift from Prof. Gubbels, Boston College) at a dilution of 1:1000, and acetylated α-tubulin (Santa Cruz Biotechnology) at a dilution of 1:200.

### RNA sample preparation and extraction

RNA samples were prepared by infecting HFF cell monolayers in T175 flasks with iKD TgAP2XII-9 parasites for 24 hours, followed by a 2 or 6-hour treatment with auxin before collecting the samples and adding Trizol (Invitrogen). Control samples were cultured in regular media. RNA extraction was conducted according to the manufacturer’s instructions, followed by removal of genomic DNA and purification using the RNase-free DNase I Amplification Grade Kit (Sigma). The quality of all RNA samples was assessed using an Agilent 2100 Bioanalyzer, with only samples having an integrity score of 8 or higher included in the RNA library preparation. 5 biological replicates were generated for Auxin treated conditions and 3 biological replicates were generated for control conditions.

### RNA library preparation and validation

RNA libraries were prepared using the TruSeq Stranded mRNA Sample Preparation Kit (Illumina), following the manufacturer’s protocol. Validation of the libraries was performed using DNA high-sensitivity chips on an Agilent 2100 Bioanalyzer. Quantification of the libraries was conducted using quantitative PCR (12K QuantStudio).

### RNA-sequencing analysis

Bcl2fastq 2.17 (Illumina) was utilized for demultiplexing. The quality of the dataset was assessed using FastQC v0.11.8–0, while adapter treatment for sequencing was performed using Cutadapt v1.18. Trimmomatic v0.39 was employed to filter out reads shorter than 30 bp and those with low-quality bases. Following data cleaning, alignment against the *T*. *gondii* ME49 genome from ToxoDB was carried out using HiSAT2 v2.2.1. Gene expression quantification was performed on annotated genes using htseq-count from the HTseq suite v1.99.2. Differential gene expression analysis was conducted using DeSeq2 v1.22.1, with P-values adjusted using the Benjamin-Hochberg method. Gene expression exhibiting a fold change >2 or < -2 and an adjusted P value < 0.01 was deemed significantly differentially expressed.

### Western blotting

Western blot analysis was conducted by cultivating 2x10^6 parasites of the iKD TgAP2XII-9 strain in regular media overnight, followed by the addition of auxin for durations of 30 minutes, 1, 2, and 6 hours. Control samples were left to grow in normal media. Parasite samples were harvested by filtration and subsequent centrifugation. The resulting pellet was re-suspended in a loading buffer composed of 240 mM Tris-HCl pH 6.8, 8% SDS, 40% sucrose, 0.04% bromophenol blue, and 400 mM DTT. This was followed by denaturation through incubation of the parasite samples at 95°C for 10 minutes. Protein extracts were separated by electrophoresis on an 8% polyacrylamide gel and then transferred onto a nitrocellulose membrane (GE Healthcare) for 90 minutes at 100V. To block the membrane, a blocking buffer containing 5% milk in TBS buffer comprising 100 mM Tris pH 8, 150 mM NaCl, and 0.1% Tween was employed. The Western blot membranes were then subjected to incubation with primary antibodies for 1 hour, followed by four washes and an additional hour of incubation with secondary antibodies. Super Signal West Femto Maximum Sensitivity Substrate (Thermo Scientific) was utilized to visualize protein bands, with ChemiDocTM XRS+ (Biorad) employed for band visualization. The antibodies used included anti-HA, anti-Ty, anti-Myc, and anti-TgMIC3, each at a dilution of 1:1000, 1:500, 1:500, and 1:400, respectively. The secondary antibody utilized was species-specific and conjugated to HRP.

### Cleavage under targets and tagmentation (CUT & Tag)

CUT&Tag was employed to identify the genomic localization of TgAP2XII-9. For each sample, intracellular parasites cultured for 24 hours were harvested from a T-175 flask, lysed using a syringe, filtered through a 3 μm filter, and quantified. A total of 20 million (2 × 10^7) parasites were centrifuged at 2,000 × g for 10 minutes, and the resulting pellets were directly processed using the CUT&Tag-IT Assay Kit (Active Motif 53160).

Indexed libraries for each sample were evaluated using Agilent Bioanalyzer, pooled, and sequenced on a NovaSeq6000 to generate paired-end reads. The reads were demultiplexed using bcl2fastq version 2.20.0 and processed with cutadapt v3.4 to eliminate sequencing adapters from the 3’ end of reads, discarding any reads with less than 30 base pairs. The remaining reads were aligned to version 64 of the *Toxoplasma gondi*i ME49 reference obtained from ToxoDB using HISAT2 v2.2.1. For each sample, peaks were called using the callpeak command within MACS2. Overlapped peaks across the three biological replicates were determined using the bedtools overlap function. Peak annotation was conducted with CHIPSeeker (an R package) employing a 2-kb cutoff distance, and the peaks were finally annotated against version 64 of the *T*. *gondii* reference genome in ToxoDB.

## Supporting information

S1 Fig iKD TgAP2XII-9 and iKD TgAP2III-2 mutant construction and TgAP2III-2 is dispensable for parasite proliferation *in vitro*. **(A)** Illustration of strategy used to construct the iKD mutants of TgAP2XII-9 and TgAP2III-2. A CRISPR/Cas9-assisted homologous recombination was used to generate the iKD strains, in which the endogenous TgAP2XII-9 and tgAP2III-2 is tagged with an AID domain, HA tag and HXGPRT selection cassette. **(B)** PCR verification of the integration of the HXGPRT-HA-AID cassette at the correct genomic locus of the iKD TgAP2XII-9 **(i)** and TgAP2III-2 **(ii)** mutant. A band corresponding to 2776bp and 2045bp using iKD TgAP2XII-9 and iKD TgAP2III-2 genomic DNA respectively, confirms cassette integration, compared to the absence of this band using Tir1(WT)genomic DNA. A positive control was used to confirm the presence of the genomic DNA. **(C)(i)** Plaque assay depicting the proliferation and growth of the iKD AP2III-2 and Parental strains in presence and absence of Auxin. **(C)(ii)** Quantification of the number of lysis plaques in the iKD TgAP2III-2 and parental strain reveals the non-essentiality of TgAP2III-2. Statistical analysis was performed using a two-tailed Student’s t-test, with significance indicated by ns>0.05. Data are presented as mean ± s.d. (n  =  3).
(TIF)

S2 Fig Plastid and Golgi segregation in the iKD AP2XII-9 throughout the tachyzoite stage. **(A)** IFA and confocal imaging depicting iKD TgAP2XII-9 parasites labelled plastid (red) and Golgi (green) in the presence and absence of overnight Auxin treatment. The IFA revealed no segregation defects in Golgi and plastid. **(B)(i)** Bar graph representing the ratio of Golgi: nucleus using the parental and iKD TgAP2XII-9 strains in the absence and presence of overnight auxin treatment. A Student’s *t*-test was performed, significance denoted by ns>0.05; mean ± s.d. (*n*  =  3). **(B)(ii)** Bar graph representing the ratio of Plastid: nucleus using the parental and iKD TgAP2XII-9 strains in the absence and presence of overnight auxin treatment. A Student’s *t*-test was performed, significance denoted by ns>0.05; mean ± s.d. (*n*  =  3).
(TIF)

S3 Fig TgAP2XII-9 and TgAP2III-2 have no combinatorial effect on the parasite biology *in vitro*. **(A) (i)** Plaque assay depicting the proliferation of the double mutant in presence and absence of auxin. **(A)(ii)** Quantification of the number of plaques in the iKD TgAP2XII-9 and Double mutant strains. A two tailed Student’s *t*-test was performed, significance denoted by *p<0.05, **p<0.01, ****p<0.0001; mean ± s.d. (*n*  =  3). (B) Growth assay for parental and iKD TgAP2XII-9, iKD TgAP2III-2 and the Double mutant strains with and without 24-hour auxin treatment. Statistical analysis was performed using a two-tailed Student’s t-test, with significance indicated by *p<0.05, ns>0.05. Data are presented as mean ± s.d. (n  =  3). **(C)** IFA and confocal imaging illustrating the multiple nuclei and IMC defect phenotype labelled by TgIMC3(red) and TgENO2(green) in the Double mutant strain in presence and absence of auxin, scale bar = 5 μm. **(D)** Quantification of the multiple nuclei phenotype in the **parental and iKD TgAP2XII-9, iKD TgAP2III-2 and the Double mutant strains with and without overnight auxin treatment.** Statistical analysis was performed using a two-tailed Student’s t-test, with significance indicated by ns>0.05. Data are presented as mean ± s.d. (n  =  3). **(E)** Quantification of the IMC defect phenotype in the parental and iKD TgAP2XII-9 and the Double mutant strains after 6 hrs. auxin treatment. Statistical analysis was performed using a two-tailed Student’s t-test, with significance indicated by ****p<0.0001, ns>0.05. Data are presented as mean ± s.d. (n  =  3).
(TIF)

S4 Fig TgAP2XII-9 regulates key genes important for daughter parasite formation. **(A)** Heatmap showing the cell cycle expression all the upregulated transcripts upon the depletion of TgAP2XII-9. Majority if the upregulated transcripts show peak expression across the cell cycle. (B) Heatmap showing the cell cycle expression all the downregulated transcripts upon the depletion of TgAP2XII-9. Majority if the downregulated transcripts show peak expression during the late S, M,C and G1 phases of the cell cycle.
(TIF)

S5 Fig TgAP2XII-9 has a different biological role from TgAP2XII-2 and MORC. **(A)** Cell cycle expression of the 25 genes that are directly regulated and targeted by MORC and TgAP2XII-9 and bound by TgAP2XII-2 at their promoters. Most of them show basal level of expression throughout the cell cycle, while some of them show expression peaks during the M, C and the G1 phase. **(B)** Heatmap of the 25 genes during the different life stages of the parasite show that these genes are preferentially expressed in the bradyzoite and sexual stages of the life cycle.
(TIF)

S6 Fig Complementation of the iKD TgAP2XII-9 demonstrate that the TgAP2XII-9 protein is responsible for the phenotypes observed in the mutant and the AP2 domain is crucial for its function. **(A)** CUT & Tag track s of 3 replicates of TgAP2XII-9-HA and Tir1 strains showing the targeting of TgAP2XII-9 to its own promoter suggesting a negative feedback loop. **(B)** IFA and confocal imaging illustrating the localisation of the complemented TgAP2XII-9 to the nucleus in presence of auxin when the native TgAP2XII-9-HA is depleted. Endogenous TgAP2XII-9 tagged with HA is represented in red while exogenous TgAP2XII-9 tagged with myc is represented in green. Scale bar = 5 μm. **(C)** Bar graph representing the expression of TgAP2XII-9 using anti-HA and anti-myc antibodies in the complemented strain. mean ± s.d. (n = 3 independent experiments). **(D)** Quantification of the IMC defect phenotype in the iKD-C TgAP2XII-9 and iKD TgAP2XII-9 strains. Statistical analysis was performed using a two-tailed Student’s t-test, with significance indicated ns>0.05. Data are presented as mean ± s.d. (n  =  3). **(E)** IFA and confocal imaging illustrating the localisation of the complemented TgAP2XII-9 to the nucleus in presence of auxin when the native TgAP2XII-9-HA is depleted. Endogenous TgAP2XII-9 tagged with HA is represented in red while exogenous TgAP2XII-9 tagged with myc is represented in green. Scale bar = 5 μm. **(D)** IFA and confocal imaging illustrating the localisation of the complemented TgAP2XII-9ΔAP2 to the nucleus in presence of auxin when the native TgAP2XII-9-HA is depleted. Endogenous TgAP2XII-9 tagged with HA is represented in red while exogenous TgAP2XII-9ΔAP2 tagged with myc is represented in green. Scale bar = 3 μm.
(TIF)

S1 Table List of genes significantly affected 2 hours after the depletion of TgAP2XII-9. (XLSX)

S2 Table List of genes significantly affected 6 hours after the depletion of TgAP2XII-9. (XLSX)

S3 Table List of genes that are upregulated in both 2-hour and 6-hour transcriptomic data following the depletion of TgAP2XII-9. (XLSX)

S4 Table List of genes that are down-regulated in both 2-hour and 6-hour transcriptomic data following the depletion of TgAP2XII-9. (XLSX)

S5 Table List of genes associated with peaks identified by CUT&Tag experiments conducted with three biological replicates. (XLSX)

S6 Table List of genes present in both transcriptomic and CUT&Tag datasets to identify direct targets of TgAP2XII-9. (XLSX)

S7 Table List of primers used in this study. (XLSX)
