# Supplementary figures and images for: Cascading expression of ApiAP2 transcription factors controls daughter cell assembly in Toxoplasma gondii
Source: PLoS Pathog. 2024 Dec 30;20(12):e1012810. doi: 10.1371/journal.ppat.1012810 (PMC11723607; doi:10.1371/journal.ppat.1012810)

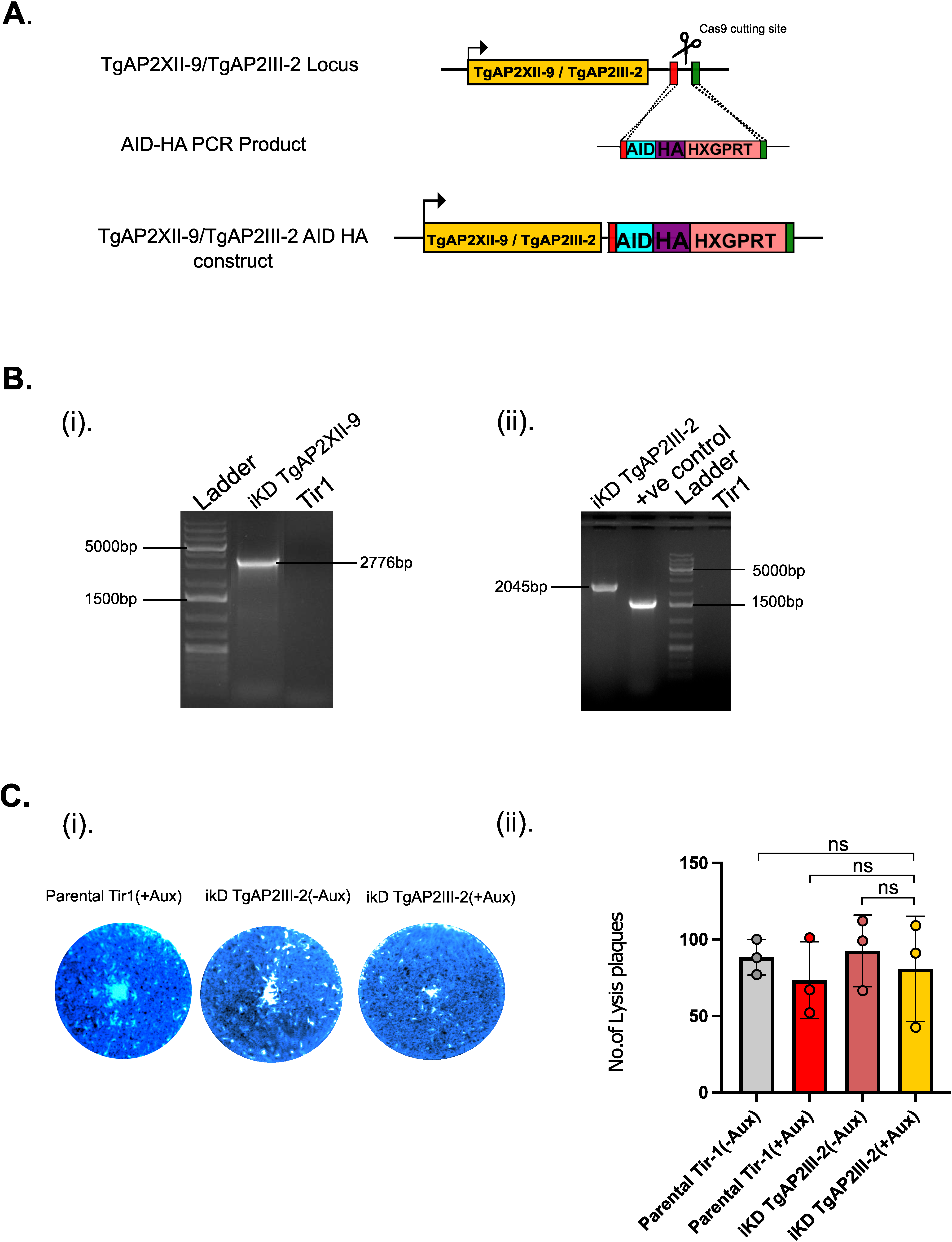

Supplement: S1 Fig — (A) Illustration of strategy used to construct the iKD mutants of TgAP2XII-9 and TgAP2III-2. A CRISPR/Cas9-assisted homologous recombination was used to generate the iKD strains, in which the endogenous TgAP2XII-9 and tgAP2III-2 is tagged with an AID domain, HA tag and HXGPRT selection cassette. (B) PCR verification of the integration of the HXGPRT-HA-AID cassette at the correct genomic locus of the iKD TgAP2XII-9 (i) and TgAP2III-2 (ii) mutant. A band corresponding to 2776bp and 2045bp using iKD TgAP2XII-9 and iKD TgAP2III-2 genomic DNA respectively, confirms cassette integration, compared to the absence of this band using Tir1(WT)genomic DNA. A positive control was used to confirm the presence of the genomic DNA. (C)(i) Plaque assay depicting the proliferation and growth of the iKD AP2III-2 and Parental strains in presence and absence of Auxin. (C)(ii) Quantification of the number of lysis plaques in the iKD TgAP2III-2 and parental strain reveals the non-essentiality of TgAP2III-2. Statistical analysis was performed using a two-tailed Student’s t-test, with significance indicated by ns>0.05. Data are presented as mean ± s.d. (n = 3). (TIF) [file ppat.1012810.s001.tif]

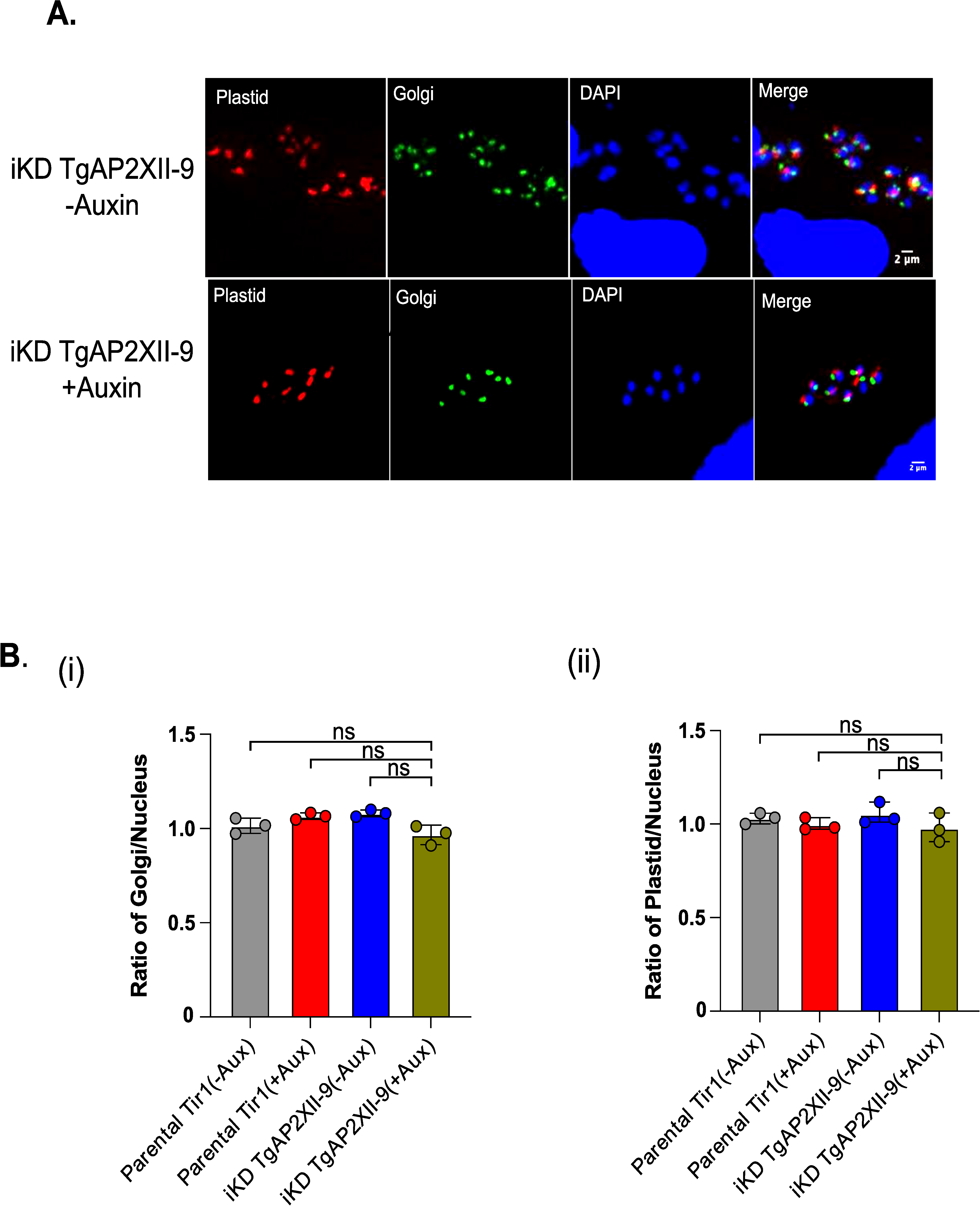

Supplement: S2 Fig — (A) IFA and confocal imaging depicting iKD TgAP2XII-9 parasites labelled plastid (red) and Golgi (green) in the presence and absence of overnight Auxin treatment. The IFA revealed no segregation defects in Golgi and plastid. (B)(i) Bar graph representing the ratio of Golgi: nucleus using the parental and iKD TgAP2XII-9 strains in the absence and presence of overnight auxin treatment. A Student’s t-test was performed, significance denoted by ns>0.05; mean ± s.d. (n = 3). (B)(ii) Bar graph representing the ratio of Plastid: nucleus using the parental and iKD TgAP2XII-9 strains in the absence and presence of overnight auxin treatment. A Student’s t-test was performed, significance denoted by ns>0.05; mean ± s.d. (n = 3). (TIF) [file ppat.1012810.s002.tif]

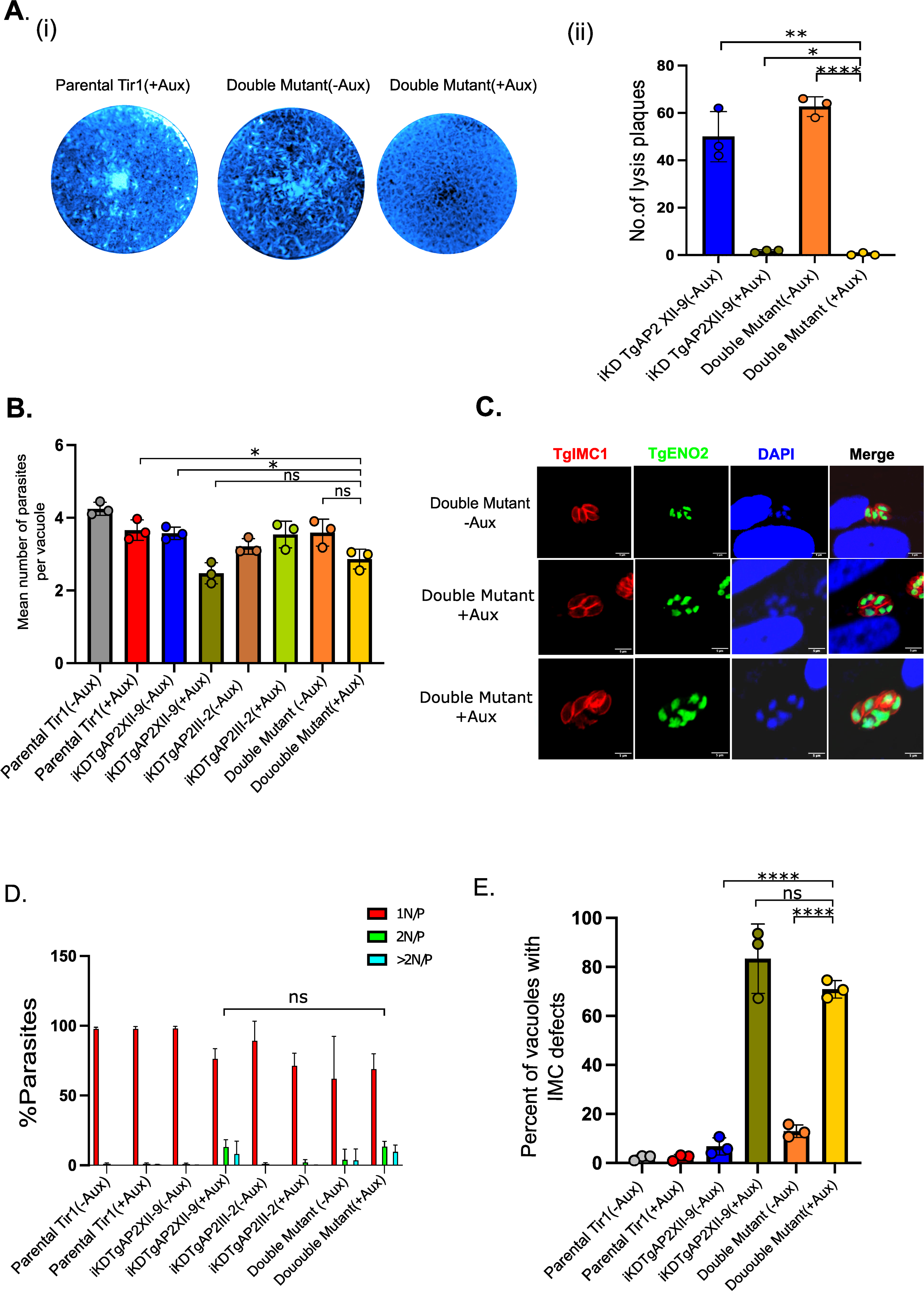

Supplement: S3 Fig — (A) (i) Plaque assay depicting the proliferation of the double mutant in presence and absence of auxin. (A)(ii) Quantification of the number of plaques in the iKD TgAP2XII-9 and Double mutant strains. A two tailed Student’s t-test was performed, significance denoted by *p<0.05, **p<0.01, ****p<0.0001; mean ± s.d. (n = 3). (B) Growth assay for parental and iKD TgAP2XII-9, iKD TgAP2III-2 and the Double mutant strains with and without 24-hour auxin treatment. Statistical analysis was performed using a two-tailed Student’s t-test, with significance indicated by *p<0.05, ns>0.05. Data are presented as mean ± s.d. (n = 3). (C) IFA and confocal imaging illustrating the multiple nuclei and IMC defect phenotype labelled by TgIMC3(red) and TgENO2(green) in the Double mutant strain in presence and absence of auxin, scale bar = 5 μm. (D) Quantification of the multiple nuclei phenotype in the parental and iKD TgAP2XII-9, iKD TgAP2III-2 and the Double mutant strains with and without overnight auxin treatment. Statistical analysis was performed using a two-tailed Student’s t-test, with significance indicated by ns>0.05. Data are presented as mean ± s.d. (n = 3). (E) Quantification of the IMC defect phenotype in the parental and iKD TgAP2XII-9 and the Double mutant strains after 6 hrs. auxin treatment. Statistical analysis was performed using a two-tailed Student’s t-test, with significance indicated by ****p<0.0001, ns>0.05. Data are presented as mean ± s.d. (n = 3). (TIF) [file ppat.1012810.s003.tif]

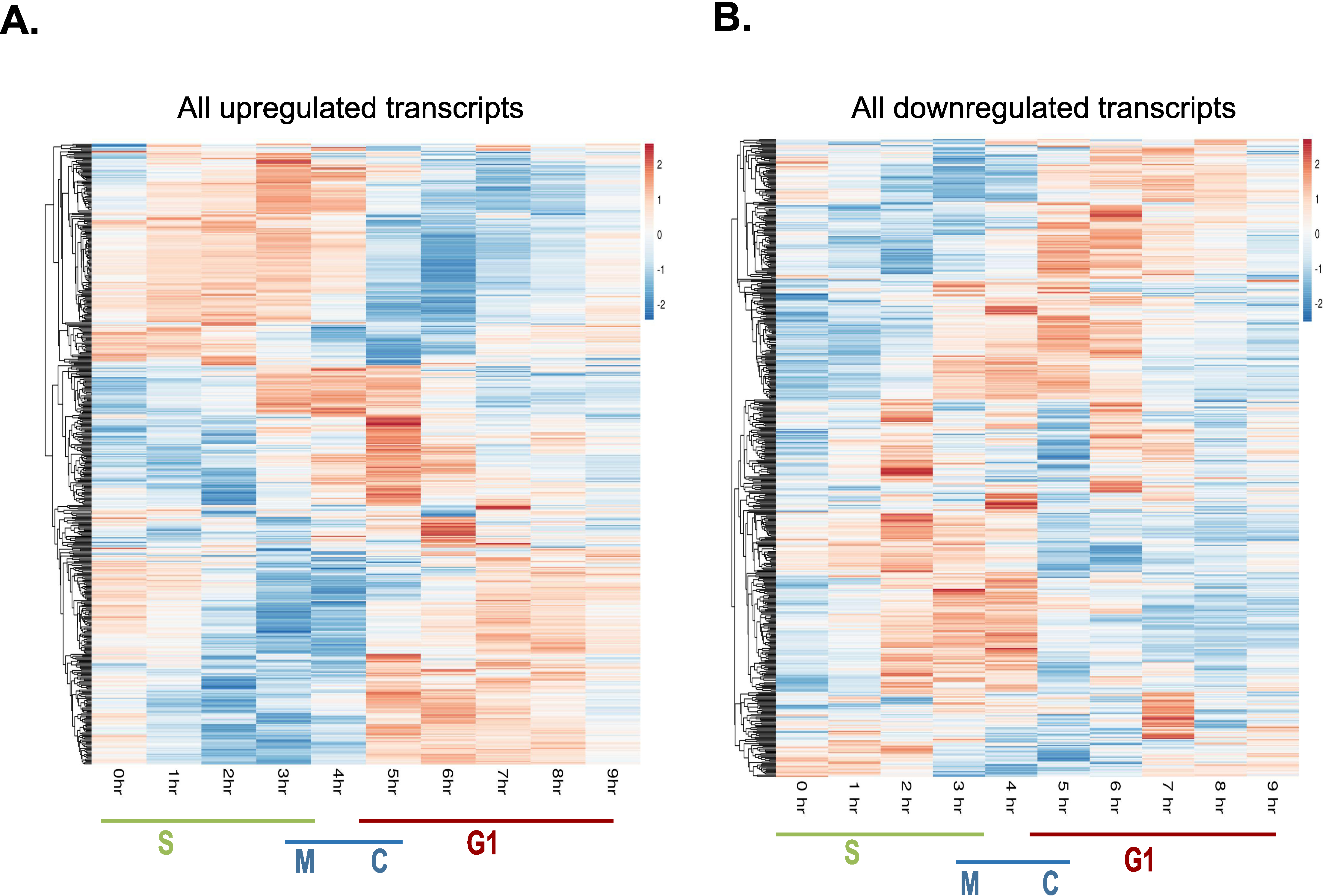

Supplement: S4 Fig — (A) Heatmap showing the cell cycle expression all the upregulated transcripts upon the depletion of TgAP2XII-9. Majority if the upregulated transcripts show peak expression across the cell cycle. (B) Heatmap showing the cell cycle expression all the downregulated transcripts upon the depletion of TgAP2XII-9. Majority if the downregulated transcripts show peak expression during the late S, M,C and G1 phases of the cell cycle. (TIF) [file ppat.1012810.s004.tif]

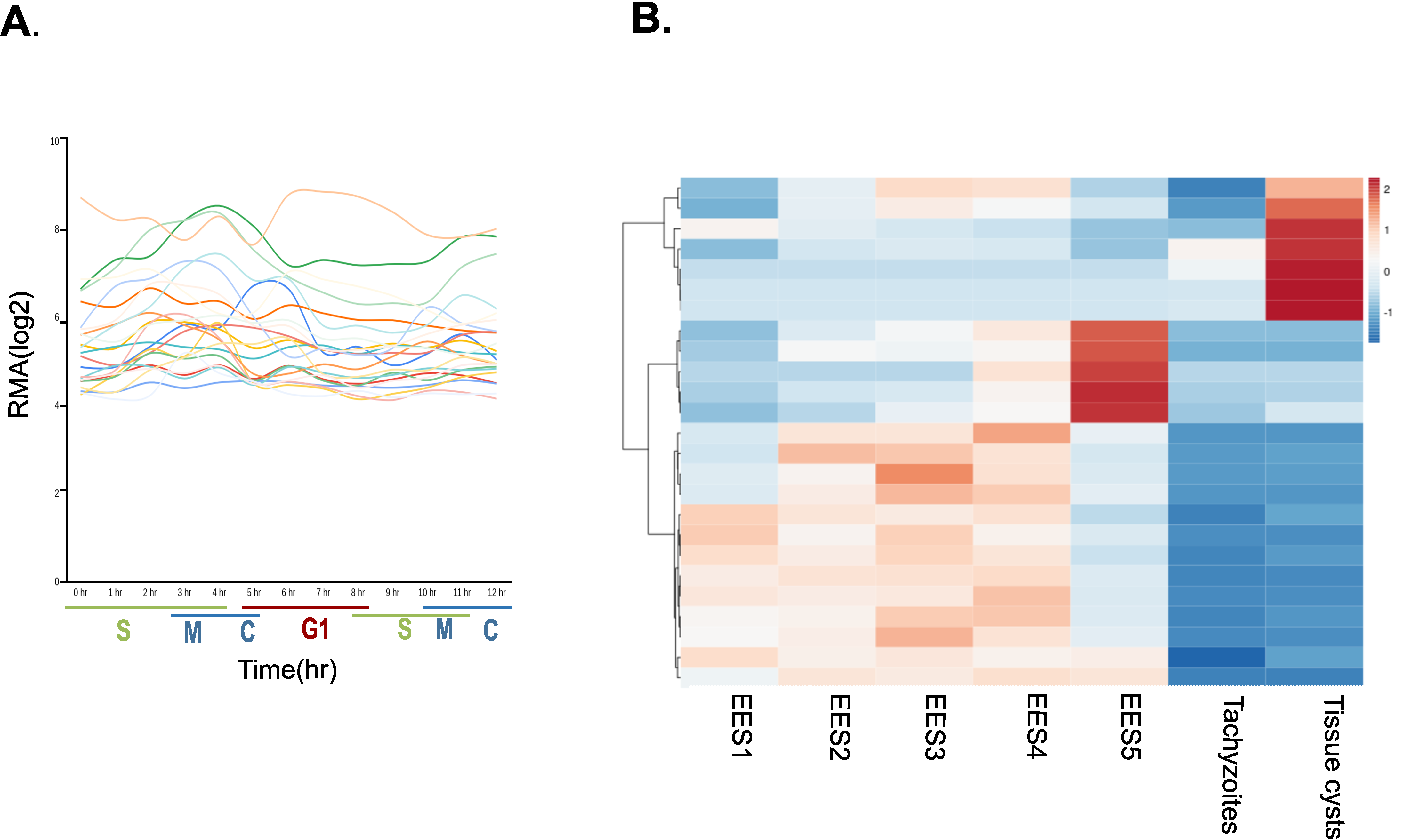

Supplement: S5 Fig — (A) Cell cycle expression of the 25 genes that are directly regulated and targeted by MORC and TgAP2XII-9 and bound by TgAP2XII-2 at their promoters. Most of them show basal level of expression throughout the cell cycle, while some of them show expression peaks during the M, C and the G1 phase. (B) Heatmap of the 25 genes during the different life stages of the parasite show that these genes are preferentially expressed in the bradyzoite and sexual stages of the life cycle. (TIF) [file ppat.1012810.s005.tif]

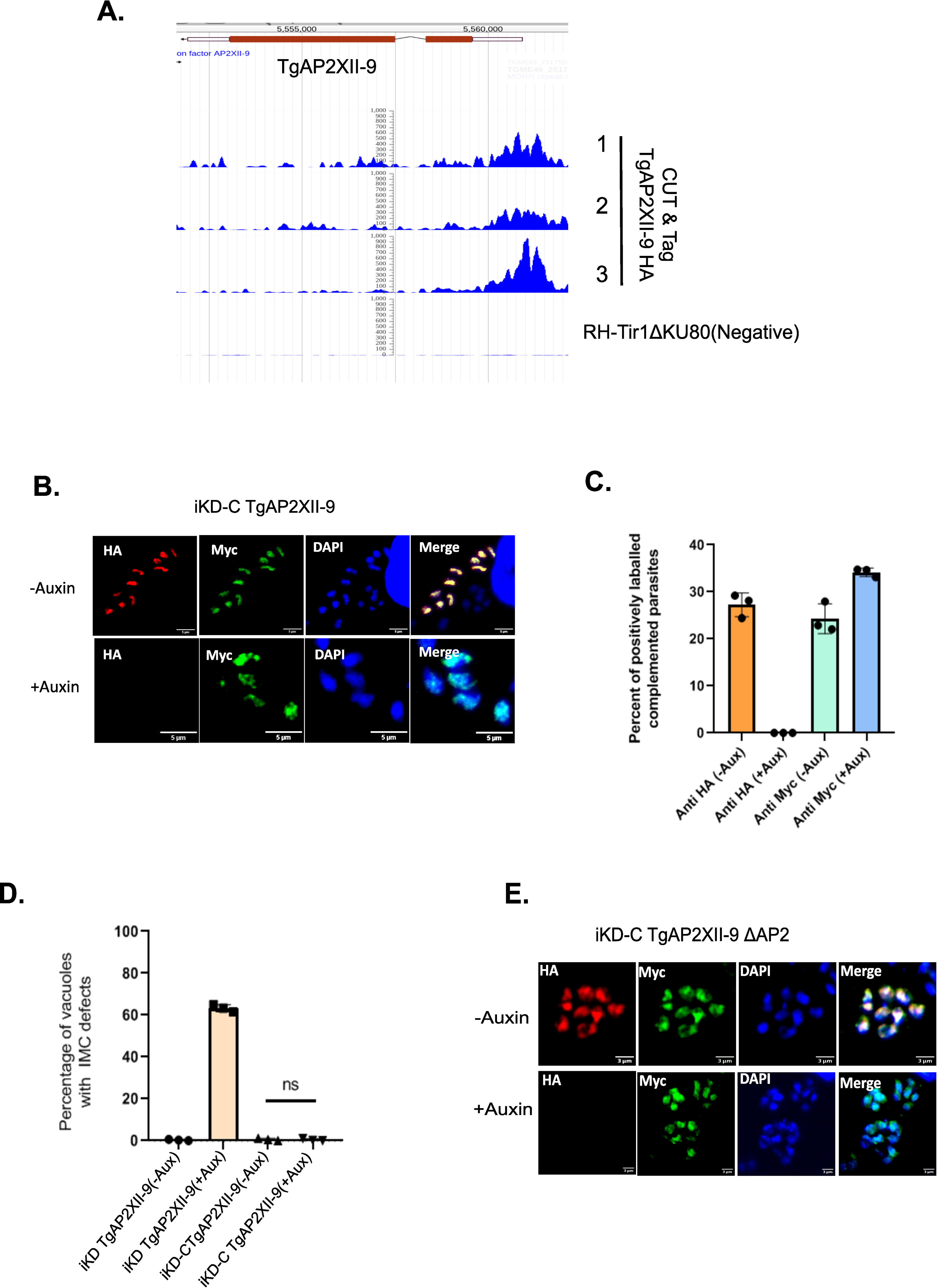

Supplement: S6 Fig — (A) CUT & Tag track s of 3 replicates of TgAP2XII-9-HA and Tir1 strains showing the targeting of TgAP2XII-9 to its own promoter suggesting a negative feedback loop. (B) IFA and confocal imaging illustrating the localisation of the complemented TgAP2XII-9 to the nucleus in presence of auxin when the native TgAP2XII-9-HA is depleted. Endogenous TgAP2XII-9 tagged with HA is represented in red while exogenous TgAP2XII-9 tagged with myc is represented in green. Scale bar = 5 μm. (C) Bar graph representing the expression of TgAP2XII-9 using anti-HA and anti-myc antibodies in the complemented strain. mean ± s.d. (n = 3 independent experiments). (D) Quantification of the IMC defect phenotype in the iKD-C TgAP2XII-9 and iKD TgAP2XII-9 strains. Statistical analysis was performed using a two-tailed Student’s t-test, with significance indicated ns>0.05. Data are presented as mean ± s.d. (n = 3). (E) IFA and confocal imaging illustrating the localisation of the complemented TgAP2XII-9 to the nucleus in presence of auxin when the native TgAP2XII-9-HA is depleted. Endogenous TgAP2XII-9 tagged with HA is represented in red while exogenous TgAP2XII-9 tagged with myc is represented in green. Scale bar = 5 μm. (D) IFA and confocal imaging illustrating the localisation of the complemented TgAP2XII-9ΔAP2 to the nucleus in presence of auxin when the native TgAP2XII-9-HA is depleted. Endogenous TgAP2XII-9 tagged with HA is represented in red while exogenous TgAP2XII-9ΔAP2 tagged with myc is represented in green. Scale bar = 3 μm. (TIF) [file ppat.1012810.s006.tif]
